# Gene Annotation and Drug Target Discovery in *Candida albicans* with a Tagged Transposon Mutant Collection

**DOI:** 10.1371/journal.ppat.1001140

**Published:** 2010-10-07

**Authors:** Julia Oh, Eula Fung, Ulrich Schlecht, Ronald W. Davis, Guri Giaever, Robert P. St. Onge, Adam Deutschbauer, Corey Nislow

**Affiliations:** 1 Department of Genetics, Stanford University, Palo Alto, California, United States of America; 2 Stanford Genome Technology Center, Palo Alto, California, United States of America; 3 Department of Pharmaceutical Sciences, University of Toronto, Toronto, Ontario, Canada; 4 Banting and Best Department of Medical Research and Department of Molecular Genetics, University of Toronto, Toronto, Ontario, Canada; 5 Donnelley Center for Cellular and Biomolecular Research, Toronto, Ontario, Canada; 6 Physical Biosciences Division, Lawrence Berkeley National Lab, Berkeley, California, United States of America; 7 Virtual Institute for Microbial Stress and Survival, Lawrence Berkeley National Lab, Berkeley, California, United States of America; David Geffen School of Medicine at University of California Los Angeles, United States of America

## Abstract

*Candida albicans* is the most common human fungal pathogen, causing infections that can be lethal in immunocompromised patients. Although *Saccharomyces cerevisiae* has been used as a model for *C. albicans*, it lacks *C. albicans'* diverse morphogenic forms and is primarily non-pathogenic. Comprehensive genetic analyses that have been instrumental for determining gene function in *S. cerevisiae* are hampered in *C. albicans*, due in part to limited resources to systematically assay phenotypes of loss-of-function alleles. Here, we constructed and screened a library of 3633 tagged heterozygous transposon disruption mutants, using them in a competitive growth assay to examine nutrient- and drug-dependent haploinsufficiency. We identified 269 genes that were haploinsufficient in four growth conditions, the majority of which were condition-specific. These screens identified two new genes necessary for filamentous growth as well as ten genes that function in essential processes. We also screened 57 chemically diverse compounds that more potently inhibited growth of *C. albicans* versus *S. cerevisiae*. For four of these compounds, we examined the genetic basis of this differential inhibition. Notably, Sec7p was identified as the target of brefeldin A in *C. albicans* screens, while *S. cerevisiae* screens with this compound failed to identify this target. We also uncovered a new *C. albicans*-specific target, Tfp1p, for the synthetic compound 0136-0228. These results highlight the value of haploinsufficiency screens directly in this pathogen for gene annotation and drug target identification.

## Author Summary

*Candida albicans* is a normal inhabitant in our bodies, yet it can become pathogenic and cause infections that range from the superficial in healthy individuals to deadly in the immunocompromised. Comprehensive genetic analysis of *C. albicans* to identify mechanisms of virulence and new treatment strategies has been hampered by limited, publically accessible genomic resources. By combining the principles of *Saccharomyces cerevisiae* strain tagging with transposon mutagenesis to generate individually tagged mutants, we created the first entirely public resource that allows simultaneous measurement of strain fitness of ∼60% of the genome in a wide range of experimental treatments. By identifying genes that confer a fitness or growth defect when reduced in copy number, we uncovered genes whose protein products represent potential antifungal targets. Moreover, screening this strain collection with chemical compounds allowed us to identify anticandidal chemicals while concurrently gaining insight into their cellular mechanism of action. This resource, combined with straightforward screening methodology, provides powerful tools to generate hypotheses for functional annotation of the genome, and our results highlight the value of direct versus model-based pathogen studies.

## Introduction

Fungal species of the genus *Candida* generally live communally on and in the human body, yet *Candida* infections can become systemic and lethal in up to 60% of immunocompromised patients [1], [2]. *C. albicans* alone accounts for over 50% of all fungal infections [3]. Furthermore, drug resistance to current therapies is becoming increasingly prevalent [4], motivating research efforts to understand the genetic basis of *C. albicans*' pathogenesis and uncover novel therapeutic targets.

The etiology of *C. albicans* is particularly complex, and our understanding of this pathogen lags with respect to the model organism *Saccharomyces cerevisiae*, which is often used as a proxy for *Candida* species despite the fact that *S. cerevisiae* diverged from *C. albicans* between 150–800 million years ago [5], [6]. Notably, *S. cerevisiae* is rarely pathogenic [7] and lacks the multiple morphogenic forms that define *C. albicans* pathogenicity. *C. albicans* also exists as an obligate diploid and lacks a traditional meiotic cycle [8]; as a result, many of the genetic tools developed in *S. cerevisiae* are not easily applied. Finally, only 58% of predicted *C. albicans* proteins share an ortholog with *S. cerevisiae* [9], [10], underscoring the need for direct study of *C. albicans*.

An effective approach for such analyses would be to extend high-throughput methodologies developed in *S. cerevisiae* to *C. albicans.* For example, experimental multiplexing, in which a genome-wide collection of deletion mutants is pooled and grown competitively to determine the fitness of each mutant in an experimental condition, has been particularly effective in *S. cerevisiae* (reviewed in [11] and [12]). Strain tracking and quantitation is enabled by the presence of unique DNA sequences, or tags, introduced during the construction of each deletion mutant [13]. To measure strain abundances in pooled growth experiments, these strain-specific tags can be amplified and hybridized to a microarray containing the tag complements, or sequenced directly [14], [15]. The *S. cerevisiae* deletion collection and pooled phenotypic profiling have been invaluable for examining gene function [14], genetic interactions [16], and for the identification of drug targets and their mechanism of action [17], [18], [19], [20].

Strategies for large-scale mutant screening in *C. albicans* include two studies using transposon mutagenesis to rapidly generate large numbers of mutants [21], [22], and a third study using targeted deletions combined with a regulatable promoter [23]. While each has been used to uncover novel biological insights, several factors have limited their widespread utility to the *C. albicans* research community. For instance, mutants in one heterozygous disruption collection [22] are largely unsequenced, making predictions regarding its coverage difficult. The homozygous transposon disruption collection [21], although sequenced and archived as individual mutants, is unavailable as an entire collection and its strains are not tagged; thus experiments must be conducted individually. A proprietary tagged deletion collection has been used to identify novel antifungal targets [23], [24], [25], [26], but the composition of this collection is limited to 2868 strains that share homology to genes essential in *S. cerevisiae,* other fungi, and higher eukaryotes. Accordingly, a majority of *C. albicans*-specific genes are not interrogated.

Our goal was to create an unbiased, open-access collection of tagged *C. albicans* mutants useful for high-throughput experimental multiplexing. We previously reported the creation of a pilot pool of 1290 tagged *C. albicans* mutants, using universal “TagModules” to label transposons that were subsequently used to simultaneously generate mutants and integrate a pair of DNA tags at the insertion site [27]. Here, we describe the creation and validation of a genome-wide *C. albicans* tagged transposon mutant collection, using TagModule-based transposon mutagenesis to generate 4252 mutant strains, 3633 of which were detectable by microarray in a pooled growth assay.

To demonstrate the utility of this collection, we investigated nutrient-specific and drug-induced haploinsufficiency. By screening four different media conditions and 57 primarily synthetic inhibitory compounds, we 1) identified genes functioning in core or essential processes, 2) uncovered genes specific to growth in a particular nutrient condition, and 3) demonstrated the utility of this collection in antifungal screening to determine mechanism of action of inhibitory compounds. This collection represents a public, archived resource of tagged *C. albicans* mutants that can be used to examine gene function either individually or multiplexed in a pool.

## Results

### Construction and validation of a tagged *C. albicans* collection

**Figure 1:**
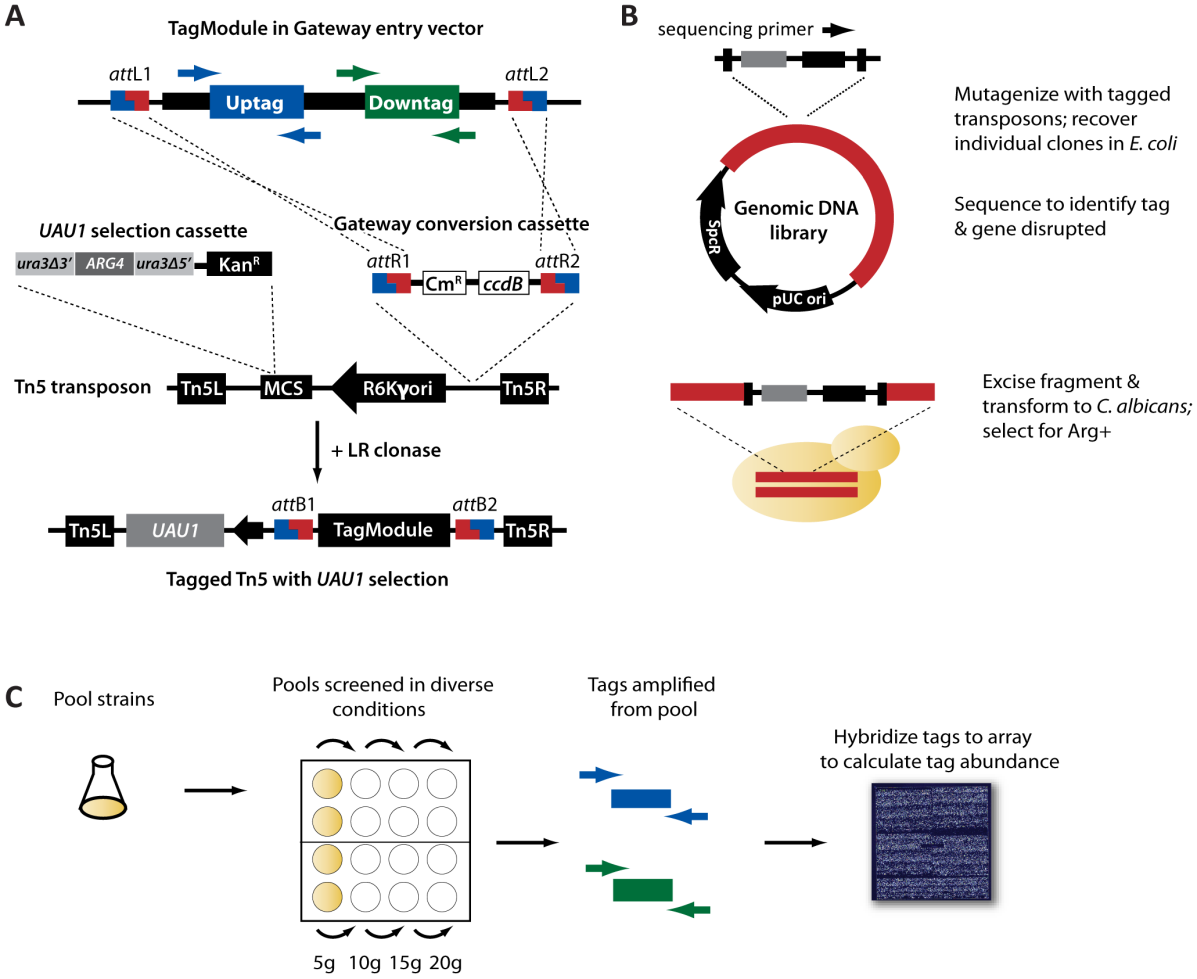
Construction of tagged transposon mutants in *C. albicans*. (A) A commercial Tn5 transposon was modified by inclusion of a Gateway conversion cassette (with the *ccdB* selection gene and chloramphenicol resistance gene (Cm^R^)), a kanamycin resistance gene (Kan^R^), and the *UAU1* marker cassette at the multiple cloning site (MCS). A reaction with the modified Tn5, a pool of TagModules, and LR clonase induces recombination at the *att* sites, placing the TagModule within the transposon mosaic ends (Tn5L and Tn5R). (B) To generate the heterozygous disruption strains, multiple genomic DNA libraries were constructed and then mutagenized *in vitro* with the pool of tagged Tn5 transposons from (A). Plasmids containing individual insertions were recovered from *E. coli* and sequenced (arrow) to determine the disrupted gene and its corresponding tag. Results were sorted to maximize the unique gene insertions/tag pairs. Select insertions were amplified, followed by excision of the genomic DNA containing the tagged transposon insertion. Transformation of these inserts into *C. albicans* was mediated by homology of the genomic sequence flanking the transposon insertion, and integration was selected for based on the arginine prototrophy conferred by the Arg+ marker. Homologous recombination results in a gene disruption with a tagged Tn5 transposon. (C) Screening methodology. Approximately equivalent numbers of tagged transposon mutants are combined to create a tagged pool. Pools are then screened in diverse conditions, generally over 20 generations of competitive growth with pools being harvested and diluted every 5 generations (5 g, 10 g, 15 g, 20 g). The genomic DNA is then extracted from the pool and the uptags and downtags amplified separately using the common priming sites. After the pooled growth assay, the amplicons are then hybridized to an Affymetrix TAG4 microarray to calculate tag intensity as a proxy for strain abundance.

To circumvent the resource-intensive approach of generating gene-specific deletion cassettes to knock out gene function, we used tagged transposon mutagenesis to generate mutants. To incorporate tags into the transposon, we used 4280 Gateway-compatible TagModules developed in a previous study as a source of sequence-verified tags [27]. Each TagModule contains a pair of DNA tags, an uptag and a downtag, flanked by common priming sites for amplification of the tags to determine strain abundance. These TagModules were transferred to a Gateway-compatible transposon modified to contain the *UAU1* selectable marker, which allows heterozygous (Arg+) as well as homozygous disruption mutants (Arg+, Ura+) to be generated [28]. The design of the tagged transposons is shown in Figure 1A.

Following the approach of previous transposon mutagenesis studies [21], [22], these tagged transposons were used to mutagenize a *C. albicans* genomic library *in vitro*. Following *in vitro* mutagenesis, unique insertions were recovered in *E. coli* and sequenced individually to determine the disrupted gene and its linked TagModule (Figure 1B). We recovered a total of 21,468 usable insertion events (see Methods S1 for criteria) representing 4827 unique genes (∼78% of predicted open reading frames). 4239 of these were successfully transformed (for criteria, see Methods) into *C. albicans* via homologous recombination to create a uniquely tagged, heterozygous disruption mutant (Table S2).

**Figure 2:**
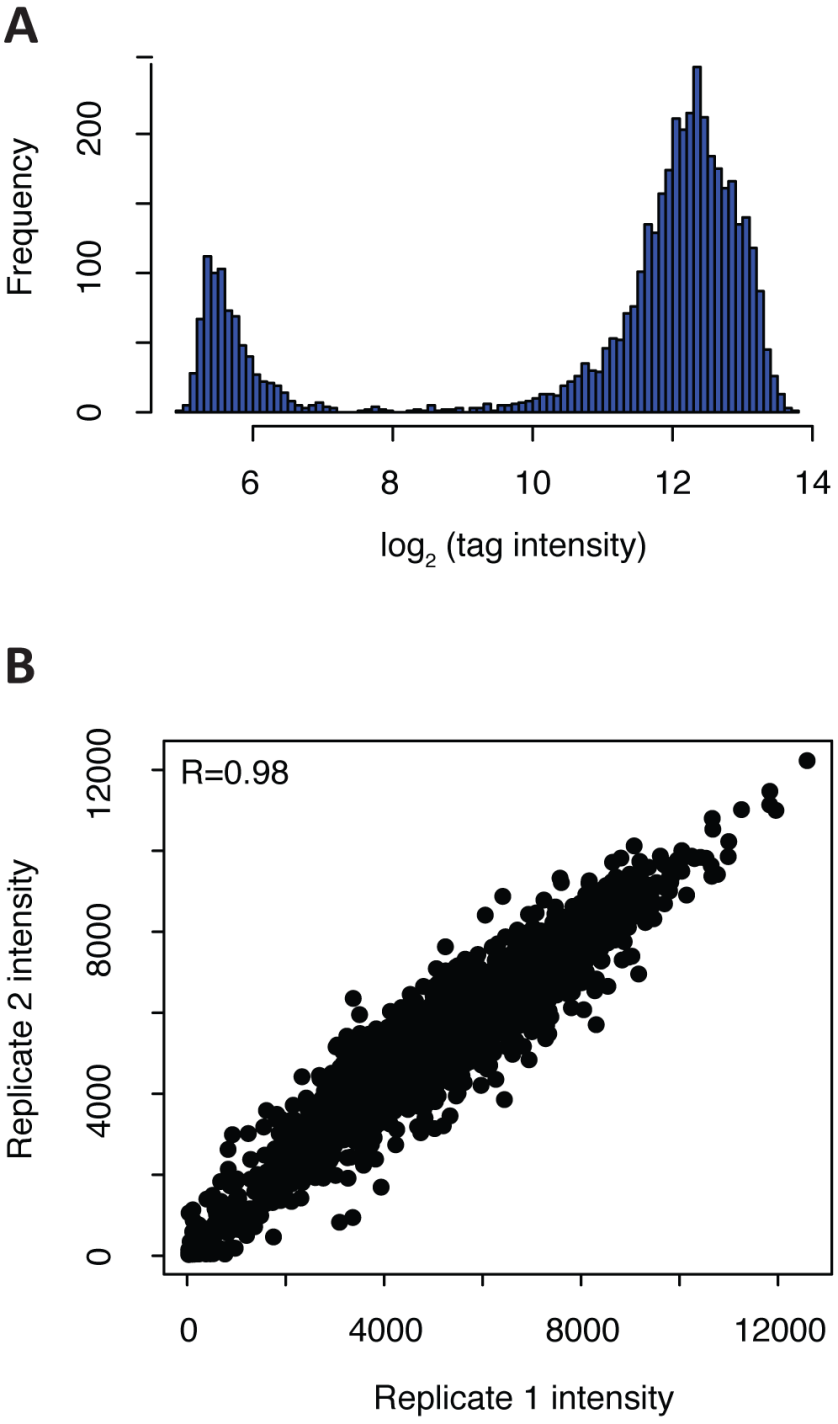
Validation of tagged *C. albicans* mutants via microarray hybridization. (A) Distribution of log_2_ tag intensities of the 4239 successfully transformed strains when pooled and tags amplified and hybridized to a TAG4 array. Log_2_ of background intensity  =  ∼6; cutoff for detection was 3X background (log_2_  =  ∼7). 3633 strains were detected with microarray signal intensity 3X above background. (B) Biological replicates of *C. albicans* pool are highly correlated even when using one tag per strain. Independent pools representing the 4239 heterozygous disruption strains were grown for 20 generations in YPD +1% DMSO. Tags were amplified from each pool and hybridized to a TAG4 array. Pearson correlation of unnormalized tags is indicated in the upper left.

To examine the quality of our collection, we pooled the 4239 strains, amplified their tags, and hybridized them to an Affymetrix TAG4 array (Figure 1C). At a zero timepoint (i.e., with no competitive outgrowth), 3633 strains were detected (tag intensity above 3X median background), with 619 strains falling below background (Figure 2A). Failure to detect strains may result from either a failure of the transformant to re-grow, or low abundance prior to pooling. If the latter, these strains could assayed as a separate subpool, or alternatively, detected using high-throughput sequencing, which provides increased sensitivity [15]. After twenty generations of growth in a pooled assay, biological replicates were highly correlated (Figure 2B; R = 0.98, p<10^−16^) and strains showed low cross-reactivity with other features on the array; the vast majority (12292/12686, or 97%) of unused tags on the array had signal intensity below our cutoff of 3X background (Figure S3). Thus, our tagged transposon mutants have robust and reproducible performance in a pooled format.

### Nutrient-dependent haploinsufficiency profiling of tagged mutants

Genes that have a growth defect when reduced in copy number from two to one (termed haploinsufficiency) are of interest for their potential as drug targets [12]. We therefore sought to use the pooled growth assay to define genome-wide haploinsufficiency in *C. albicans*, particularly for *C. albicans*-specific processes and those required for growth in hyphae-inducing conditions, a determinant of virulence. We screened the *C. albicans* pool in four different nutrient conditions at 30°C: i) rich YPD media, a standard laboratory growth condition, ii) a synthetic media used for the selection of transformants (SC), iii) minimal media, which has been used to induce formation of pseudohyphae and consists of 2% glucose and yeast nitrogen base (YNB), and iv) a low-nitrogen minimal media (synthetic low ammonium dextrose, or SLAD), which can induce pseudohyphal/hyphal growth in *C. albicans*.

To assess the effect of each gene disruption on growth in these conditions, we tracked tag abundance for each of the detectable 3633 strains, assaying after five, ten, fifteen, and twenty generations of growth with biological replicates. Following calculation of tag abundance by hybridization of amplified tags to a microarray, we fitted a linear regression to each strain's abundance over a timecourse and used the slope of the regression to measure strain sensitivity. Based on Deutschbauer et al. [29], we defined a strain as having a growth defect if its slope was <0, p<0.05 (Table S3).

**Figure 3:**
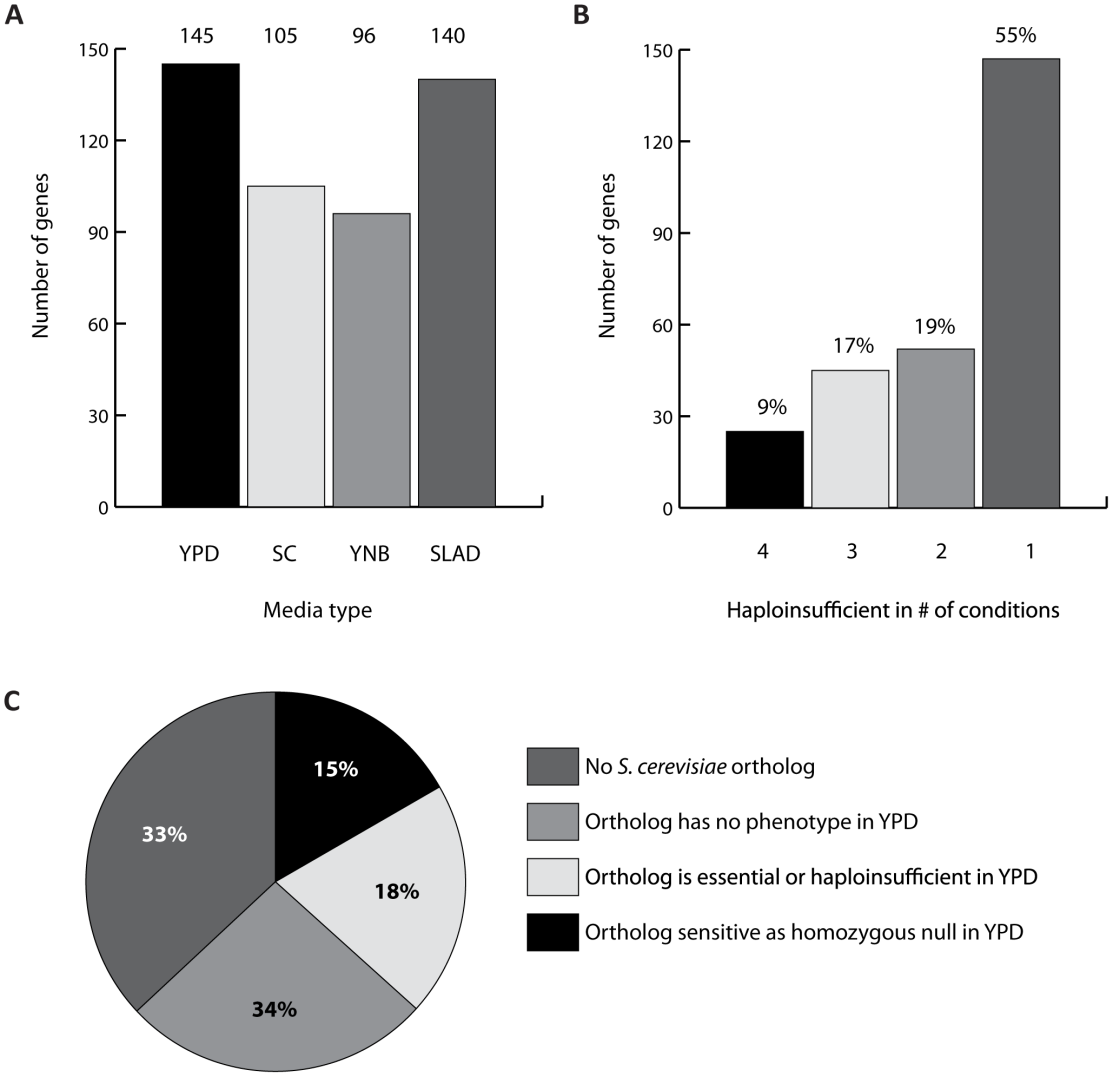
Using the pooled growth assay to examine haploinsufficiency in *C. albicans*. (A) Pooled growth of 3633 strains was measured in rich (YPD), semi-rich (SC), minimal (YNB), and low-nitrogen (SLAD) media. Samples were collected every 5 generations, after which tags were amplified and hybridized to an array. A linear regression was fitted to the log_2_ of the normalized tag intensity values as a function of generation time. Strains were categorized as haploinsufficient if the regression slope of their time course was statistically significantly less than 0 (adjusted p<0.05). The total number of haploinsufficient strains was determined for each media condition. (B) The 269 haploinsufficient strains from the four media conditions were separated into bins depending on whether they showed haploinsufficiency in one, two, three, or all media conditions. (C) Comparison between *C. albicans* and *S. cerevisiae* haploinsufficient genes in rich media. Orthologs were retrieved for the 145 haploinsufficient genes in YPD (http://www.candidagenome.org/), then compared against results from haploinsufficiency profiling of *S. cerevisiae* in YPD [29].

We found that regardless of media condition, similar proportions of strains were haploinsufficient (Figure 3A). Overall, 145 (4%) strains were haploinsufficient in rich YPD, 105 (2.9%) in SC, 97 (2.7%) in YNB, and 140 (3.9%) in the low-nitrogen SLAD, representing 269 (7.4%) unique genes. Only 9% (25) of these genes were haploinsufficient in all conditions; the majority (55%) of these 269 genes were haploinsufficient in a single condition, suggestive of condition-dependent haploinsufficiency (Figure 3B). This observation suggests that a substantial portion of the *C. albicans* genome may be amenable to haploinsufficiency profiling under certain conditions.

Finally, comparing our haploinsufficiency profiling data in rich media to that of *S. cerevisiae* [29], we found that while the overall proportion of haploinsufficient strains was similar, we found a number of biological differences (Figure 3C). 48/145 (33%) of *C. albicans* haploinsufficient genes in YPD had no *S. cerevisiae* ortholog. Of those *C. albicans* genes that did have an ortholog, 26 are orthologous to *S. cerevisiae* genes that were essential or haploinsufficient in YPD, and an additional 22 orthologs exhibited a growth defect as a homozygous deletion in YPD. 49 (34%) orthologs had no phenotype in YPD. Of the 269 haploinsufficient genes in *C. albicans*, 97 (36%) had no *S. cerevisiae* ortholog. A second striking difference between the *S. cerevisiae* and *C. albicans* haploinsufficient gene sets is the number of transcription factors (TFs) haploinsufficient in *C. albicans*. Few transcription factors (excluding general regulatory factors) were haploinsufficient in *S. cerevisiae* [29]. In contrast, we identified seven TFs as haploinsufficient in YPD (*ZCF9*, *HAC1*, *LYS142*, *IRO1*, *SUA71*, *BDF1*, *RBF1*), and a total of 13 in the complete haploinsufficient dataset (*FGR17*, *SEF1*, *SUA72*, *orf19.6623*, *orf19.5368*, *MSN4*). Their functions range from iron utilization to regulators of filamentous growth or stress response. These findings highlight the need for direct study of *C. albicans*, as our assay uncovers novel phenotypes for orthologs as well as a large number of *C. albicans-*specific genes.

To search for functional enrichment among the 25 genes haploinsufficient in all conditions (Table S4), we used the Gene Ontology Term Finder from the Candida Genome Database (http://www.candidagenome.org/cgi-bin/GO/goTermFinder). These genes were significantly enriched for the GO processes “translation” and GO functions “structural constituent of ribosome” and “structural molecule activity” (Table 1). We also observed genes in this set that function in “core” cellular processes that are likely to be either essential or necessary for growth regardless of condition; for example, *POL2* (DNA polymerase epsilon) and *orf19.736*, whose *S. cerevisiae* ortholog Sc-*SRB8* is a subunit of RNA polymerase II required for transcriptional regulation [30]. We also identified a putative permease (*orf19.3293*), which may play a role in nutrient sensing, two genes whose products protect against oxidative stress (*SOD3* and *orf19.5553*, based on *S. cerevisiae* orthology), and a putative 5′-monophosphate 5′-nucleotidase, *ISN1*, which is a fungal-specific gene whose *S. cerevisiae* ortholog has been implicated in nucleotide scavenging [31]. Interestingly, the *S. cerevisiae* orthologs for these four genes are not haploinsufficient, suggesting that *S. cerevisiae* and *C. albicans* have diverged with respect to the relative importance of these genes for survival, or that these genes are required at greater than heterozygote gene doses in *C. albicans*.

**Table 1:** GO analysis of genes haploinsufficient in 3 or more media types.

# of conditions	GO analysis	GO ID	Go term	Cluster frequency	Background frequency	Corrected p-value	Gene(s) annotated to the term
4	process	6412	translation	7/25 genes, 28.0%	374/6435 genes, 5.8%	0.03	RPS26A:RPS10:RPS1:RPL32:RPL43A:RPS30:TIF35
3+	process	51029	rRNA transport	4/70 genes, 5.7%	33/6435 genes, 0.5%	0.09	RPS26A:RPS10:NUP188:RPS15
3+	process	6407	rRNA export from nucleus	4/70 genes, 5.7%	33/6435 genes, 0.5%	0.09	RPS26A:RPS10:NUP188:RPS15
3+	function	3735	structural constituent of ribosome	9/70 genes, 12.9%	161/6435 genes, 2.5%	0.00	RPS26A:RPS10:RPL10:RPS1:RPS21B:RPL32:RPL43A:RPS30:RPS15
3+	function	5198	structural molecule activity	10/70 genes, 14.3%	270/6435 genes, 4.2%	0.03	RPS26A:RPS10:RPL10:RPS1:RPS21B:RPL32:RPL43A:RPS30:NUP188:RPS15
3+	function	146	microfilament motor activity	2/70 genes, 2.9%	6/6435 genes, 0.1%	0.09	orf19.230:MYO2

Because the 25 genes that were haploinsufficient in all conditions (“4 or more” subset) were enriched for genes involved in fundamental cellular processes, we propose that the unverified genes in this set (those which lack annotation, or those whose annotation has not been confirmed experimentally) are also involved in core or essential processes. As the GO enrichments for genes haploinsufficient in 3 or more conditions (Table S5) were similar to the 4 or more subset (Table 1), we expanded our list of “core” genes to include these genes as well, for a total of 70 genes. Consistent with our observations in the 4 or more subset, this 3 or more group included additional permeases (*MUP1* and *orf19.5826*) and two genes with predicted involvement in oxidative stress response (*TRR1* and *POS5*).

### Assigning function to the core haploinsufficient set by complementation

To ask if we could assign functions to this unverified “core” gene set, we selected 17 genes whose *S. cerevisiae* orthologs (or if an ortholog was not found, the best BLAST hit was used) are essential, so we could assess function by complementation testing in *S. cerevisiae*. Although an imperfect test for *C. albicans* function (and susceptible to false negatives), positive results strongly suggest functional similarity.

**Figure 4:**
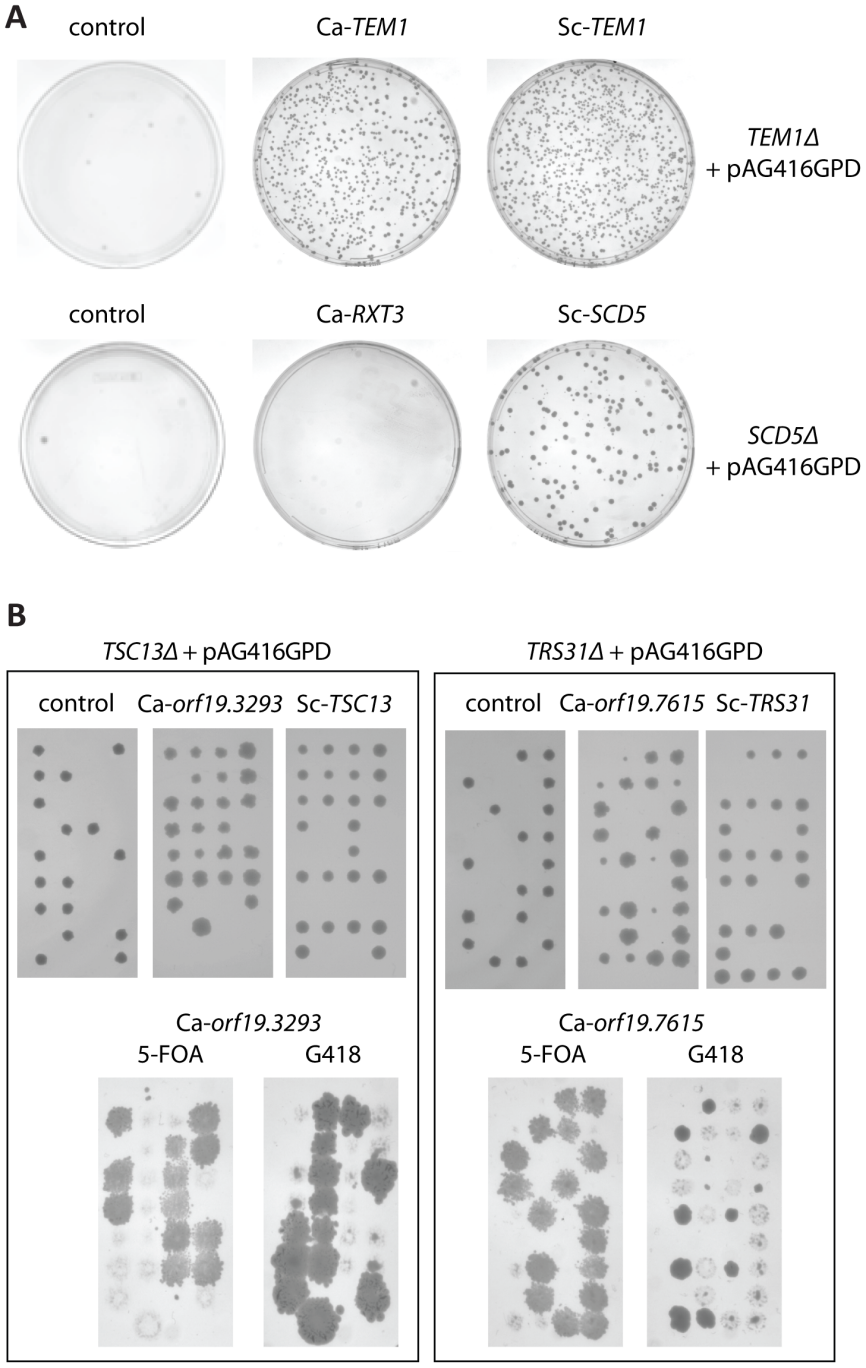
Representative results from complementation testing of “core” haploinsufficient strains. (A) Each *C. albicans* ORF was cloned into an *S. cerevisiae* CEN/ARS expression vector with expression controlled by the constitutive GPD promoter (pAG416GPD, [60]). Overexpression clones were then transformed into the corresponding *S. cerevisiae* strain containing a deletion of the essential ortholog and a Magic Marker cassette [32]. The Magic Marker cassette allowed selection of null haploid mutants via sporulation followed by selection for matA cells containing a knockout. 4.5×10^−4^ OD_600_ of sporulated culture was plated. Left, negative control (vector-only); center, complementation with *C. albicans* ORF; right, positive control (complementation with corresponding *S. cerevisiae* ORF). *TEM1* (upper panel) is an example of complementation of the *C. albicans* ORF; *RXT3* (lower panel) does not complement. (B) Selected results from (A) were verified by tetrad dissection. For each box: left, negative control (vector-only); center, positive control (complementation with corresponding *S. cerevisiae* ORF), and right, complementation with *C. albicans* ORF. The left box is an example of full complementation, and on the right, a case of partial complementation. Tetrads were then replica-plated onto media containing 5-fluoroorotic acid (5-FOA) or geneticin (G418) to confirm that the overexpression clone was the source of complementation.

We used two approaches to test for complementation. First, we cloned these 17 *C. albicans* ORFs into a CEN/ARS vector with a constitutive promoter and transformed them into the corresponding *S. cerevisiae* heterozygous mutants (YKO). Sporulation of the heterozygous deletion strain followed by selection for a haploid knockout should yield no growth unless complemented by the plasmid-borne *C. albicans* gene. To generate homozygous null *S. cerevisiae* knockouts, we used the Magic Marker strains [32], which use sequential selections to generate haploid deletion mutants. 6/15 *C. albicans* ORFs with available corresponding Magic Marker strains showed complementation, as did 9/13 *S. cerevisiae* ORFs tested as a control. Complementation was assessed based on a significant increase in the number of colonies on the overexpression plate versus the vector-only plate (Figure 4A, Figure S5).

Second, we confirmed all negative Magic Marker results by tetrad dissection (Figure 4B, Figure S5) and found that a total of 10/17 *C. albicans* ORFs (two additional YKOs were available for this test) and 13/13 *S. cerevisiae* ORFs complemented their corresponding deletion allele. In instances in which we observed complementation, segregation of spore viability improved to 3∶1 or 4∶0 (from 2∶2 in controls), indicating rescue of one or both of the haploid null spores. 3∶1 segregants likely result from incomplete segregation of the CEN/ARS-based plasmid, which generally exists in one or more copies per cell, to all four spores. Interestingly, we also observed two small-size spores in the case *orf19.7615*/Sc-*TRS31*, indicating partial complementation. Based on these results, we conclude that 10/17 *C. albicans* ORFs have the same (or very similar) cellular roles as their *S. cerevisiae* ortholog and propose the following changes to the description of these ORFs: a change of “Feature Type” from “uncharacterized” to “verified”, and a transfer of function from the *S. cerevisiae* description (summarized in Table 2).

**Table 2:** Summary of individual strain testing of 17 “core” function genes & proposed annotations.

Magic Marker complemented	Tetrad dissection complemented	#CUG codons	*C. albicans* gene	*S. cerevisiae* ortholog (gene)	Verified?	*C. albicans* description
no	no	0	MYO2	MYO2	Verified	Class V myosin
no	yes	1	orf19.3293	TSC13	Verified*	Protein similar to enoyl reductase for fatty acid elongation*
yes	NA	4	orf19.6908	FOL3	Verified*	Protein similar to dihydrofolate synthetase*
no	yes (partial)	1	orf19.7615	TRS31	Verified*	Protein similar to a TRAPP complex of the cis-Golgi*
NA	no	3	POL2	POL2	Uncharacterized	Protein described as DNA polymerase epsilon
no	no	0	RPL10	RPL10	Uncharacterized	Putative ribosomal protein
no	yes (partial)	1	RPL32	RPL32	Verified*	Protein component of the large (60S) ribosomal subunit*
no	no	0	RPS15	RPS15	Uncharacterized	Putative ribosomal protein
no	no	5	RXT3	SCD5	Uncharacterized	Predicted ORF in Assemblies 19, 20 and 21
no	no	2	SGD1	SGD1	Uncharacterized	Predicted ORF in Assemblies 19, 20 and 21
NA	yes	0	SMX4	LSM3	Verified*	Protein similar to Lsm3 involved in RNA processing and decay*
yes	yes	1	TEM1	TEM1	Verified*	Protein similar to *S. cerevisiae* Tem1p*
yes	NA	0	TIF34	TIF34	Verified	Protein similar to eIF3i subunit of translation initiation factor 3*
no	no	0	TIF35	TIF35	Uncharacterized	Putative translation initiation factor
yes	yes	0	TRR1	TRR1	Verified*	Protein similar to thioredoxin reductase*
yes	NA	8	TSR1	TSR1	Verified*	Protein similar to 20S pre-rRNA processing unit*
yes	NA	17	UGP1	UGP1	Verified	Protein similar to UTP-glucose-1-phosphaturidyl transferase

*proposed.

### Confirmation of haploinsufficient phenotypes

Finally, to test whether this group of “core” genes tested via complementation also represents essential *C. albicans* genes, we obtained GRACE strains [33] for 12/17 of the “core” group genes. Testing the GRACE strains, which are conditional heterozygous knockouts that can be converted (functionally) into homozygous knockout mutants by repression of the second allele with doxycycline, served two purposes: first, to determine whether these strains are viable as homozygotes, and second, to validate that the phenotypes of our mutants represent true positives by validating growth defects in an alternative strain background. This is particularly relevant because it is possible that synthetic effects with BWP17′s uracil or histidine auxotrophies could contribute to the observed growth phenotypes.

We examined the growth of these 12 GRACE strains as well as our 17 transposon-derived mutants in selective media. Overall, after 15–20 generations of growth, the majority of our transposon mutants recapitulated a growth defect as observed in the pooled growth assay (Figure S4A). While the growth phenotypes of the GRACE mutants were generally less severe (Figure S4B), we also observed haploinsufficiency in 11/12 of these strains (defined as <98% of BWP17′s growth rate using the metric AvgG [34]). The difference in the degree of haploinsufficiency could be the result of either a “leakiness” in the tetracycline-regulated promoter of the GRACE strains [33] causing production of additional gene product and thereby alleviating the growth phenotype, or could reflect some contribution of synthetic interactions with the histidine and uridine auxotrophies mentioned above.

When the GRACE strains were grown in the presence of 100 µM doxycycline, 10/12 (*FOL3* and *TIF34* GRACE mutants excepted) showed a severe to complete growth defect in all media types, suggesting that they are essential in *C. albicans* (Figure S4C). The fact that two strains were not essential under the GRACE test but were able to complement their essential *S. cerevisiae* ortholog could be the result of promoter leakiness, or could reflect that these genes, while capable of performing a similar function as their *S. cerevisiae* orthologs, are not essential in *C. albicans*.

### Analysis of condition-dependent haploinsufficiency

We noted that haploinsufficiency in *C. albicans* is primarily condition-dependent; 55% of the 269 haploinsufficient genes had a growth defect in a single condition, and 19% of the 269 were identified in only two conditions. Examining these genes, we found two general categories. The first category, identified in rich media, includes genes involved in oxidative metabolism, such as *COX2*, *NAD5*, *ABC1*, *KGD2*, and (by annotation transfer from *S. cerevisiae* orthologs) *orf19.4204/*Sc-*PET123*. We also observed haploinsufficiency in the *C. albicans*-specific alternative oxidases *AOX1* and *AOX2* (GO:0016682, n = 2, 1.4% vs 0% in the genome; hypergeometric p = 0.046), which are thought to function in maintaining turnover of the TCA cycle, relieving saturation in oxidative metabolism [35].

In reduced nutrient conditions, we observed a class of haploinsufficient genes related to growth in low nitrogen. Genes in this category, outlined in Table 3, were involved in 1) cell wall maintenance (*FAT1*, *SIM1*, *UPC2*), 2) nutrient sensing/acquisition (*PUT4*, *JEN2*, *GPX2*, *MEU1*, *FUR1*, *SEF1*), and 3) pseudohyphal growth regulators (*GRR1*, *FGR17*, *CPP1*). *orf19.1617*, an uncategorized gene in both *C. albicans* and *S. cerevisiae* (Sc-*YDR282C*), has also been shown to have a filamentation defect [22]. Because these categories encompass the roles of nutrient scavenging, initiation of filamentation, and cell wall remodeling necessary to produce hyphae, we asked if filamentation may be one mechanism by which *C. albicans* thrives in low-nutrient conditions.

**Table 3:** Categories of genes haploinsufficient in reduced-nutrient conditions.

Category	Gene	Description
cell wall maintenance	*FAT1*	Predicted enzyme of sphingolipid biosynthesis
	*SIM1*	Protein involved in cell wall maintenance
	*UPC2*	Transcriptional regulator of ergosterol biosynthetic genes and sterol uptake
nutrient sensing/acquisition	*PUT4*	Putative proline permease
	*JEN2*	A carboxylic acid transporter that regulates uptake of malate and succinate, which are additional sources of acetyl-CoA for the TCA cycle
	*GPX2*	Putative glutathione peroxidase with antioxidant properties
	*MEU1*	Putative enzyme in methionine salvage
	*FUR1*	Enzyme of pyrimidine salvage
	*SEF1*	Putative transcription factor regulating iron uptake
pseudohyphal growth regulators	*GRR1*	Negative regulator of yeast-pseudohyphae switch
	*FGR17*	Putative transcription factor regulating filamentous growth
	*CPP1*	MAPK regulator of filamentous growth

**Figure 5:**
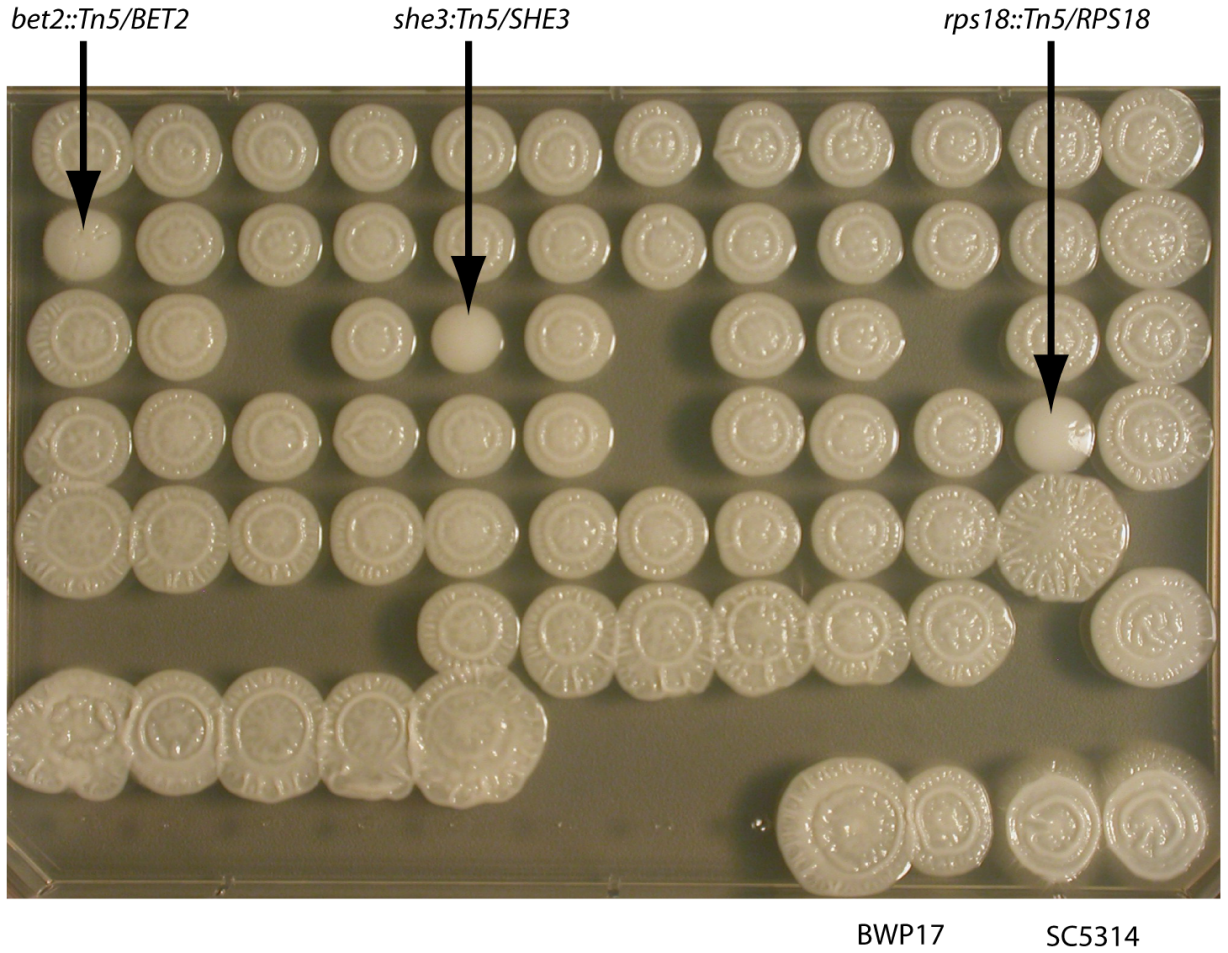
Few strains haploinsufficient in pooled growth have defects in filamentation. Strains defined from pooled screens as haploinsufficient in reduced nutrient conditions (YNB or SLAD) media, but not haploinsufficient in rich media (YPD or SC) were grown individually on agar media that induces filamentation (Spider media). Duplicates of control wild-type *C. albicans* strains BWP17 and parent strain SC5314 are in the bottom right; strains with a filamentous defect are indicated with an arrow.

We investigated if these defects in growth rate correlate with defects in filamentation by testing if the 77 genes necessary for growth in the nutrient-limiting conditions (Table S6) manifested a filamentous defect. Only 3/77 (*BET2*, *SHE3*, and *RPS18*) mutants had a distinct filamentous defect on Spider agar (Figure 5), one condition that elicits a filamentous phenotype. *SHE3* and *RPS18* had a completely smooth appearance with no peripheral filaments compared to wild-type. *BET2* had a smooth appearance with some peripheral filaments. *SHE3* has previously been shown to be necessary for filamentous growth on Spider media [36]. The other two genes (*RPS18*, a putative ribosomal protein, and *BET2*) have not previously been implicated in a filamentous phenotype. *BET2*′s *S. cerevisiae* ortholog functions in vesicle transport, and in *C. albicans BET2* is regulated by Mig1p, a transcriptional repressor that regulates carbon source utilization [37] and is downregulated in hyphal growth [38] but upregulated in biofilms [39]. While we have identified two new genes likely involved in filamentous growth, our results suggest that genes required for growth in low-nutrient conditions, as identified in our SLAD-media screens, are, in general, different from those required for filamentation in Spider agar.

We next performed a pooled screen on solid SLAD media. Briefly, ∼500,000 colonies/pool was plated onto solid SLAD agar plates and grown for 6 days at 37°C. By quantifying cells that had invaded the agarose (pellet) and comparing them to the proportions of cells that had not invaded the agar (supernatant), we were able to determine if particular mutants were defective in invasion. At a cutoff of 2-fold greater abundance in non-invaded fraction (log_2_(supernatant/pellet) >2), we observed 341 mutants that were invasion-defective (Table S8). As we previously observed, there was little overlap (5/77) between strains that were necessary for full growth in low nitrogen and those that had a defect in agar invasion. However, when we compared the 243 *S. cerevisiae* orthologs/best hits corresponding to these 341 mutants to invasion-defective mutants of the pseudohyphal *S. cerevisiae* Sigma 1278B strain (O. Ryan, personal communication), we observed a 26% overlap (62/243), underscoring 1) the ability of our pooled mutants to identify biologically consistent results, and 2) the flexibility of the pooled assay to a solid-media format. Additional experiments on solid media will provide a more detailed genome-wide perspective on this key aspect of *C. albicans* physiology.

### Drug-induced haploinsufficiency profiling

**Figure 6:**
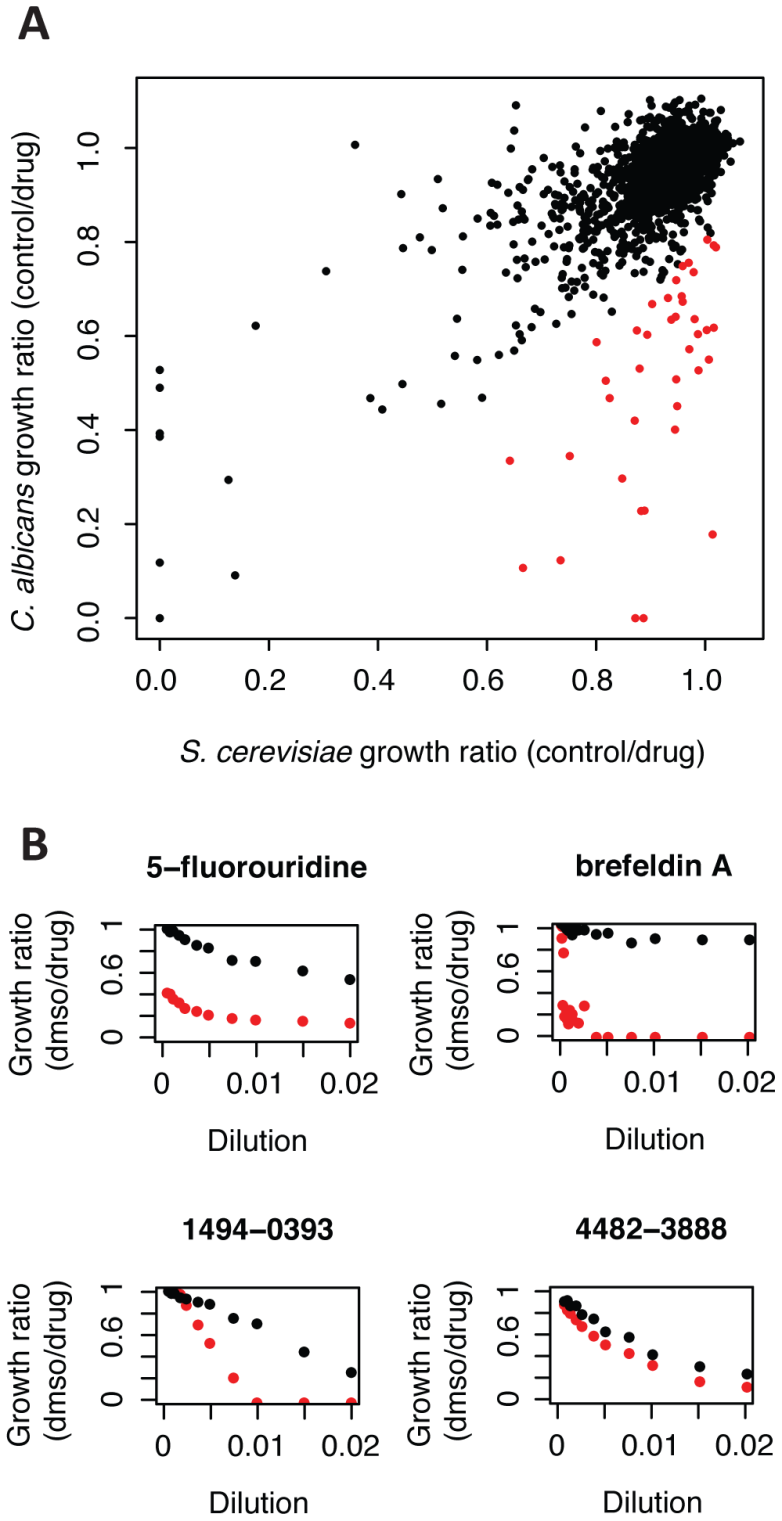
Several compounds have different inhibitory activity in *C. albicans* versus *S. cerevisiae*. (A) Comparison of compound inhibition between *S. cerevisiae* and *C. albicans*. Wild-type *S. cerevisiae* (HO-1) and wild-type *C. albicans* (BWP17) were grown in YPD +1% DMSO (control) or with one of 1521 unique chemical compounds. A growth rate ratio was calculated by averaging 12 controls per 96-well plate and dividing that by the drug-treated growth rate. Highlighted in red are 40 compounds that inhibited *C. albicans* more than *S. cerevisiae* (measured by difference in growth rate ratio). (B) Representative dose response curves for compounds with different levels of inhibition between *S. cerevisiae* and *C. albicans*. X-axis represents drug concentration/dilution: initially 2 µL of compound of stock concentration was diluted into 100 µL of YPD, then compounds were serially diluted across the plate so that the final concentration was 1/64^th^ of that at the start. Growth rate ratio is as described in (A). Wild-type *S. cerevisiae* dose response curve are in black; wild-type *C. albicans* dose response curves are in red. Compounds labeled xxxx-xxxx are synthetic, previously uncharacterized chemicals (Chemical Diversity Labs, Inc.); 5-fluorouridine and brefeldin A are well-characterized compounds.

Haploinsufficiency profiling was developed in *S. cerevisiae* to identify drugs that target gene products essential for growth, based on the premise that lowering copy number of the target gene sensitizes the corresponding heterozygous deletion strain to the drug [40]. To select compounds for screening against the tagged *C. albicans* mutants, we focused on those that inhibited *C. albicans* growth more potently than *S. cerevisiae*. We reasoned that these compounds would be more likely to have a different mechanism of action in the two yeasts, e.g., by having distinct targets, different mechanisms of influx/efflux, or different off-target effects. We screened 1521 compounds (previously titrated to a concentration that inhibits *S. cerevisiae* by ∼10%, or IC10) against wild-type *S. cerevisiae* and wild-type *C. albicans* and measured the ratio of compound-treated growth to that of a control. While we found that the majority of compounds inhibited *C. albicans* growth at levels similar to *S. cerevisiae*'s, a number of compounds inhibited *C. albicans* more severely (Figure 6A). For example, the top 40 compounds (highlighted in red) produced 20–90% greater inhibition in *C. albicans*.

From this screen, we selected for titration and pooled growth screening 67 readily available compounds that inhibited *C. albicans* more strongly than *S. cerevisiae* in our initial screen (see Methods for criteria). Comparing the dose response of these compounds for *S. cerevisiae* and *C. albicans*, we observed that the compounds that conferred differential growth inhibition fell into two classes of dose response curves (representative curves are shown in Figure 6B, Figure S6). The first class included those with parallel dose response curves, suggestive of a compound that has the same target in both yeasts but different cell permeability or residence time. A second category included those in which the dose-response curves had different slopes, suggestive of a different mechanism of action or distinct secondary effects. Overall, these results from screening a library of 1521 compounds against wild-type *C. albicans* and *S. cerevisiae* suggest that certain compounds may have distinct mechanism of action (e.g., a combination of primary and secondary activities) in *C. albicans*.

**Figure 7:**
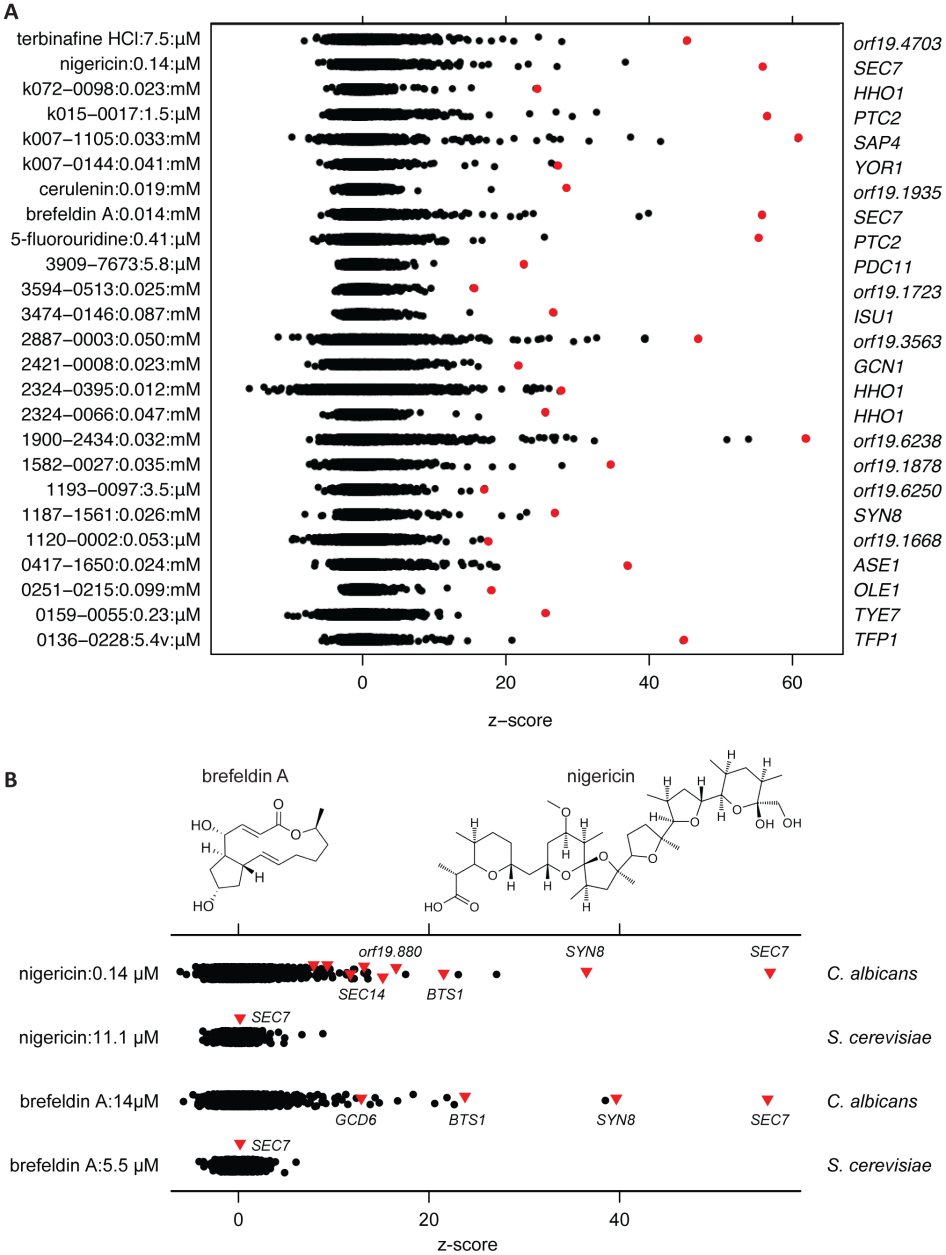
Drug-induced haploinsufficiency in *C. albicans* and profiles of vesicle transport-inhibiting compounds. (A) Comprehensive view of the fitness profiles for 25 compounds. Z-scores for each of 3573 strains were calculated by comparing the intensity values for each strain under 20 generations of compound treatment to a no-drug control set. The dot plot distribution of the z-scores is useful in identifying outliers for each compound treatment. These 25 compounds represent the 44% of those tested that resulted in a small number of significant outliers. The most sensitive strain for each screen (highlighted in red) is listed on the right. (B) Comparison of haploinsufficiency in *S. cerevisiae* and *C. albicans* for nigericin and brefeldin A. Z-scores for *S. cerevisiae* were calculated as for *C. albicans*, except only the 1150 essential heterozygotes of *S. cerevisiae* genes were screened. Top, chemical structures of these two compounds. Bottom, dotplots of the z-scores calculated for the chemical treatments, which were screened in the pooled growth assay at a concentration that produces ∼10% inhibition of wild-type. Strains marked with a red triangle are those involved in Golgi vesicle transport. Unlabeled leftmost red triangles for nigericin (*C. albicans*) represent *orf19.6558*, *orf19.2078*, and *TFP1*, *orf19.1265* (from left).

To study the genetic basis of the differential action of certain compounds in *C. albicans* and *S. cerevisiae*, we screened 57 of the 67 compounds at approximately an IC_20_ in the pooled growth assay (chemical structures are shown in Figure S7; to demonstrate that these 57 represented chemically diverse compounds, the distribution of their pair-wise similarities, based on Tanimoto scores, is shown in Figure S8). We performed a 20-generation endpoint assay (by analogy to the *S. cerevisiae* haploinsufficiency profiling) in which the abundance of each strain in a compound-treated pool was compared to its abundance in a set of DMSO-treated controls. To identify drug-induced haploinsufficiency, a normalized z-score was used to examine the response of each strain to a compound, comparing the performance of a strain (proxied by tag intensity) in the compound treatment to its performance in the no-drug controls (Table S7). Positive z-scores indicate increasing sensitivity to the treatment condition; strains with a high z-score were significantly affected by the drug treatment and may be depleted of genes that encode a cellular target. Of the 57 screens, we focused on 25 that had a small number of significantly sensitive outliers, those most likely to be representative of compounds that act through a single or small number of targets. The remaining 32 compounds exerted either widespread or few fitness defects in the pool. An overview of the z-scores for these 25 compounds is shown in Figure 7A.

We found that the GTP exchange factor *SEC7*, which activates formation of transport vesicles, was the most sensitive strain in screens with two compounds, brefeldin A and nigericin (Figure 7B). In addition, analysis of the 30 most sensitive strains from the nigericin screen in *C. albicans* showed GO enrichment of a number of vesicle transport-related processes (e.g., Golgi vesicle transport (GO:0048193), 20.7% (n = 6) versus 2.4% in the genome, hypergeometric p<0.008). As nigericin affects ion gradients across lipid membranes (as opposed to having a direct protein target [41]), we speculate that changes in membrane permeability, and by extension intracompartmental pH (see below), interact synthetically with defects in vesicle transport to cause a fitness defect. Brefeldin A, whose protein target is widely considered to be Sec7p [23] had a less pronounced effect on vesicle transport; brefeldin A-sensitive strains were GO-enriched for function “GTPase regulator activity” (GO:0005085, 6.7% (n = 2) versus 0.4% in the genome, hypergeometric p<0.07). Interestingly, neither compound induced *SEC7* haploinsufficiency in *S. cerevisiae* (Figure 7B), even though brefeldin A has been shown to inactivate *S. cerevisiae* Sec7p complexes *in vitro* [42]. These results 1) suggest that *S. cerevisiae* has additional targets that become rate-limiting for growth prior to the effects of inhibition of *SEC7*, and 2) confirm the utility of our pool for validating *C. albicans*-specific targets.

**Figure 8:**
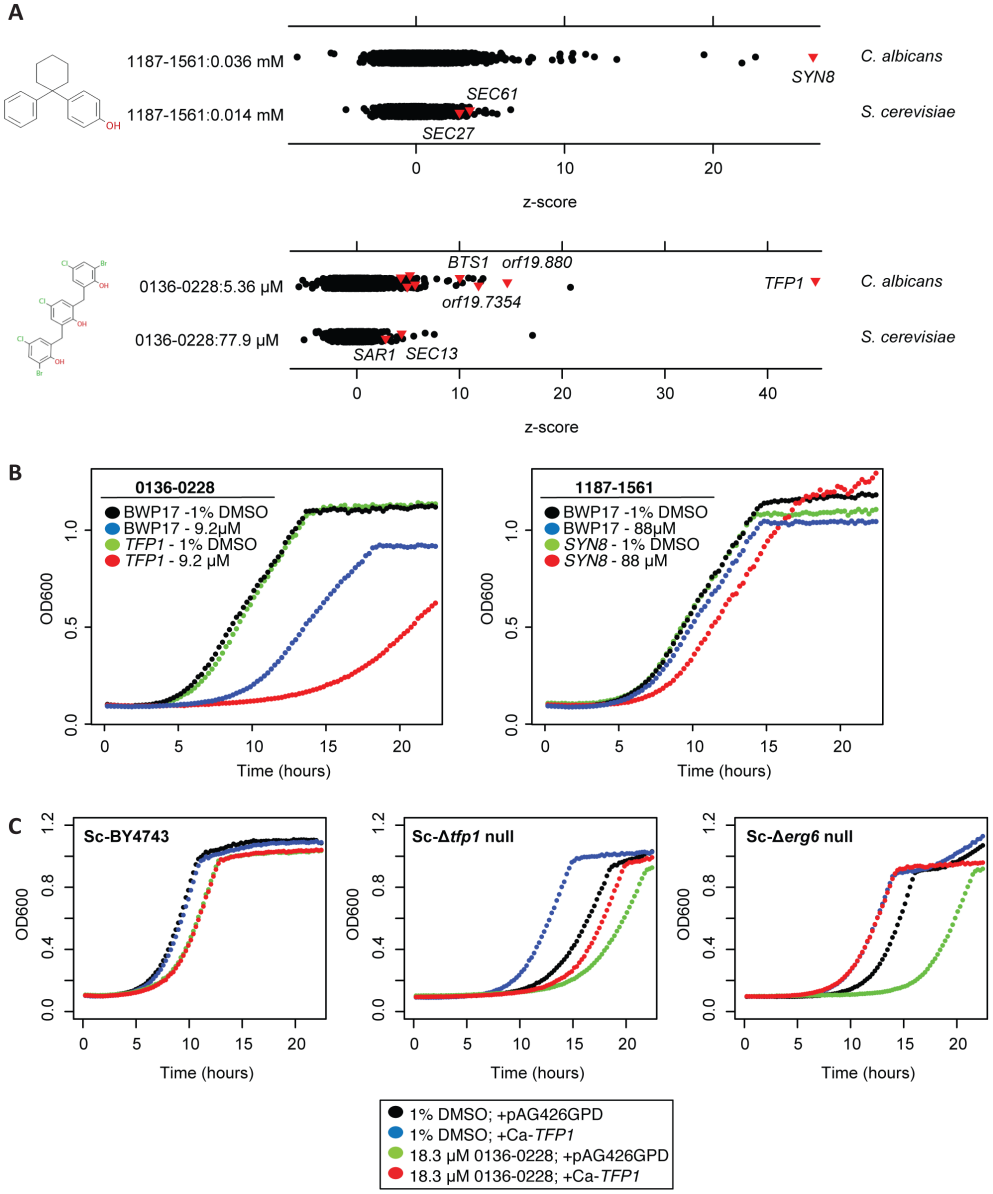
0136–0228 and 1187–1561 also inhibit vesicle transport in *C. albicans*. (A) Comparison of haploinsufficiency in *S. cerevisiae* and *C. albicans* for two Chemical Diversity Lab compounds, 1187–1561 (top) and 0136–0228 (bottom); description is as in (B). Leftmost red triangles in 0136–0228 (*C. albicans*) represent, from left, *orf19.1376*, *orf19.6558*, *orf19.2078*, and *orf19.645.1*. (B) Single strain growth curves for control (1% DMSO) and drug-treated *TFP1* (0136–0228), right, and *SYN8* (1187–1561), left. Wild-type *C. albicans* (BWP17) is included as a control for both plots (black & blue). (C) Overexpression of Tfp1p in a heterologous system alleviates growth defects resulting from treatment with 0136–0228. *S. cerevisiae* strain background is marked in the upper left corner. *TFP1* was cloned into an *S. cerevisiae* 2 micron expression vector under a constitutive GPD promoter (pAG426GPD, [60]). Overexpression clones were then transformed into one of the three *S. cerevisiae* backgrounds (wild-type BY4743, a Δ*tfp1* null, or a drug-sensitive Δ*erg6* null) and grown in the presence (18.3 µM, red) or absence (1% DMSO, blue) of 0136–0228. As a control, an empty pAG426GPD plasmid was transformed and grown in parallel (black, green).

Two synthetic, uncharacterized compounds, 1187–1561 and 0136–0228, also inhibited vesicle transport-related functions in *C. albicans* (Figure 8A). We confirmed sensitivity of these strains by growth in individual culture (Figure 8B). The most sensitive strain in a screen with 1187–1561 was *orf19.2411*::Tn5/*orf19.2411*. The *S. cerevisiae* ortholog Sc-*SYN8* (non-essential in *S. cerevisiae*) is a SNARE protein that functions in vesicle fusion [43]. 0136–0228 induced a strong growth defect in a *tfp1* mutant, which has no ortholog in *S. cerevisiae*, but is computationally predicted to encode a V-type ATPase that regulates intracompartmental pH. Because its best BLAST hit is Sc-*TFP1* and because these two genes share a fungal orthogroup [44], we surmise that they also share function. Consistent with an effect on intracompartmental pH, many of the mutants most sensitive to this compound are also sensitive to nigericin (vesicle transport related ORFs: *TFP1*, *BTS1*, *orf19.2078*, *orf19.880*, *orf19.6558*, and others: *orf19.9*, *FAA21*, *SPT5*, and *orf19.6435*). As noted above, it is likely that the genes disrupted in nigericin-sensitive strains are interacting synthetically with defects caused by altered intracompartmental pH.

We then performed a genetic test to see if 0316–0028 may interact with Tfp1p. We overexpressed *C. albicans* Tfp1p in a wild-type *S. cerevisiae* BY4743 and an Sc-Δ*tfp1* homozygous null mutant background. Because we saw no effect with wild-type *S. cerevisiae* (Figure 8C), we also tested an Δ*erg6* null mutant to account for the possibility that the compound was not penetrating the cell. Deletion of *ERG6* has been shown to increase compound penetration due to defects in the cell membrane and has been used to sensitize *S. cerevisiae* to brefeldin A [42]. First, we observed that Tfp1p overexpression ameliorated the growth defect of the Δ*tfp1* null mutant (and interestingly, also the Δ*erg6* null mutant) even in the absence of drug. This is consistent with results that show that Sc-*ERG6* and Sc-*TFP1* interact synthetically [45]. Second, when strong growth inhibition was applied with 0136–0228, we observed partial rescue of the growth defect in both the Δ*tfp1* and Δ*erg6* backgrounds, most distinctly in the drug-sensitized Δ*erg6* background (Figure 8C). That we observed rescue in a heterologous system is strong evidence that this protein is a functional target of this compound, and the fact that 0136–0228 also inhibits the Δ*tfp1* null suggests that it has additional targets in the cell. We thus propose that Tfp1p is a principal protein target of 0136–0228, and that the other sensitive strains in its chemogenomic profile appear as a secondary consequence of altered intracompartmental pH (see Discussion). Haploinsufficiency profiling in *S. cerevisiae* with these compounds would have failed to reveal these gene-compound interactions (Figure 8A).

## Discussion

Experimental multiplexing using DNA tags was one of the essential attributes of the pioneering *S. cerevisiae* deletion collection, enabling high-throughput genome annotation, genetic analysis, and antifungal discovery [14], [17], [46]. A publically available, archived, tagged mutant collection for *C. albicans* has the potential to similarly accelerate research and drug discovery in an organism directly relevant to public health. Using tagged transposon mutagenesis, we constructed a tagged *C. albicans* mutant collection that is fully sequenced identified and archived as individual mutants. We note that our collection has some caveats. First, our mutants were created in the –Arg –Ura –His strain BWP17 and so remain auxotrophic for uracil and histidine. Synthetic effects with these auxotrophies could produce false positives in some screens and follow-up assays. In such cases, complementation of *HIS1* and *URA3*, or validation of the phenotype in an alternative strain background can verify the phenotype. Our results sampling for haploinsufficiency in an alternatively constructed strain both supports that our results in BWP17-derived strains represent true positives and also suggests that there are subtle but detectable synthetic effects contributed by these auxotrophies. A second issue is that because these strains are transposon mutants, they are not likely to represent complete loss of function alleles. This latter case has the advantage that multiple insertion events with different degrees of functional disruption can be interrogated for a particular gene. The other advantage is that this approach is scalable. We report the creation of 4239 uniquely tagged gene disruptions, representing 68% of 6197 predicted ORFs. Additional mutagenesis can be used to create additional mutants, or, given their compatibility, the TagModules can be integrated into deletion cassettes to create the remaining mutants via homologous recombination.

There are many potential applications of a *C. albicans* collection; here we investigated haploinsufficiency, the phenomenon in which a single gene copy in a diploid organism results in a fitness defect. We applied the tagged mutant collection in a competitive growth assay to identify haploinsufficient genes in four different nutrient conditions, identifying 269 haploinsufficient genes across four media conditions. This dataset represents a resource for further study of their involvement in growth, morphogenesis, and potential druggable targets. We found that *C. albicans* has a unique profile of haploinsufficient genes, highlighting the importance of niche (or *C. albicans*)-specific processes for maintaining wild-type fitness. For example, *C. albicans* relies more heavily on oxidative metabolism, nutrient sensing (e.g., permeases and nutrient scavenging mechanisms), and resistance to oxidative stress for optimal growth. Consistent with this observation, the host immune response via neutrophils and macrophages involve superoxide production to kill *C. albicans* [47], suggesting that these protective mechanisms may be necessary for full growth. Moreover, dependence on oxidative metabolism is consistent with a requirement for efficient energy production for rapid growth. Because it has evolved within a human host, *C. albicans* may rely more heavily on oxidative metabolism, because carbon sources in the form of fat or proteins may be more accessible than simple sugars. Metabolites of both fat and proteins are shunted into the tricarboxylic acid (TCA) cycle as acetyl-CoA for subsequent breakdown in oxidative phosphorylation. Consistently, we found a predicted fatty acyl-coA synthetase (*FAA21*) haploinsufficient in 3 conditions. Targeting *C. albicans*-specific metabolic processes may be a useful approach for identifying novel antifungals.

We identified several transcription factors as haploinsufficient in *C. albicans*, a notable distinction from *S. cerevisiae*, suggesting that transcriptional regulation may be less flexible in *C. albicans* with heterozygous alleles manifesting haploinsufficiency. One possible explanation for this observation is that *C. albicans* is less tolerant of changes in gene dosage as it generally exists in a diploid state. In contrast, *S. cerevisiae* exists as both a haploid and a diploid, and so these changes in dosage can be tolerated without a reduction in fitness. A second possibility is that because *C. albicans* is an obligate diploid that lacks a traditional meiotic cycle, two alleles of a transcription factor have diverged such that they are no longer functionally equivalent. This is supported by extensive allelic heterozygosity observed during assembly of the *C. albicans* genomic sequence [48]. Functional allelic variation has also been observed in *C. albicans* in a number of small-scale studies, for example in drug pumps [49], or for *HWP1*, in which the two alleles are differentially expressed under biofilm conditions [50].

We next used our dataset to identify which genes function in core cellular processes, and which are haploinsufficient in a specific nutrient condition. Selecting from candidates generated through genome-wide screens, we followed up on a subset of 17 genes from a “core” set of 70 haploinsufficient genes using complementation testing and individual strain growth analysis. Although complementation testing in *S. cerevisiae* is an imperfect test of function, it is useful for generating hypotheses that can be used to infer function for the remaining uncharacterized genes in the “core” dataset of putative essential genes. We found that 10 of these 17 genes were able to complement their essential *S. cerevisiae* ortholog, strongly suggestive of functional similarity for these conserved processes. For those genes that did not complement, it is possible that their phenotypes arose from the influence of strain auxotrophies. Alternatively, failure to complement can also result from result of alternative codon usage in *C. albicans*. Interestingly, we found that both complementing and non-complementing genes contained the alternative CUG codon (Table 2)).

We also identified a set of genes necessary for growth in nutrient-limiting conditions, and found that while necessary for growth in limited nutrients, these genes were generally not necessary for filamentation. As filamentation in fungi is a well-documented response to low-nitrogen conditions (presumably an adaptation to improve nutrient acquisition) [51], we anticipated that filamentation might play a role in growth in nutrient-limited conditions. Contrary to this expectation, our results suggest a disconnect between growth rate (which may require optimal nutrient utilization) and filamentation (which may require specific nutrient sensing) under the conditions that we tested. From a biological perspective, filamentation may be the preferred lifestyle for tissue invasion or macrophage evasion in which the ability to grow rapidly is less important. *C. albicans* cells that are unable to filament *in vivo* are avirulent, and null mutants of *EFG1*, a transcriptional regulator of filamentation, have normal growth [52]. However, whether all mutants that have a filamentous defect display wild-type growth has not been systematically determined. From the standpoint of developing new treatment strategies, identifying both fungicides, which can be identified by growth inhibition screens, and inhibitors of pseudohyphal growth are of value. With the appropriate experimental design, both types of screens can be performed with this collection, as we have exemplified by preliminary results from a SLAD solid media pooled experiment.

We also investigated drug-induced haploinsufficiency in *C. albicans*, screening the pool with compounds that were most likely to produce a differential drug response by selecting compounds that more potently inhibited *C. albicans* than *S. cerevisiae*. Interestingly, we observed that the same chemical inhibitor can have different effects in *S. cerevisiae* and *C. albicans*. For instance, wild-type *S. cerevisiae* is much less sensitive to nigericin or brefeldin A than wild-type *C. albicans*, and mutants with the highest sensitivity in screens with these compounds did not include Sc-*SEC7*. This suggests that; 1) other targets in *S. cerevisiae* have a greater impact on *S. cerevisiae*'s sensitivity to brefeldin A and nigericin, 2) that the drug is detoxified in *S. cerevisiae*, or 3) that *C. albicans* has less genetic redundancy for the pathways inhibited by these compounds. We also identified two other synthetic, previously uncharacterized compounds that inhibited vesicle transport in *C. albicans*, 0136-0228 and 1187–1561. Interestingly, the sensitivity profile of 0136–0228 overlapped that of nigericin, although their chemical structures show no obvious similarity. This result can be explained if 0136–0228 inhibits Tfp1p, a putative V-type ATPase that regulates intracompartmental pH [53]. Support for this scenario came from our complementation experiments, in which the drug-induced growth defect was rescued by overexpression of Tfp1p in *S. cerevisiae*. Abrogation of intracompartmental pH regulation via disruption of ion flow across vesicle membranes likely results in growth defects similar to those produced by nigericin, which produces a similar disregulation of intracompartmental pH. Interestingly, no orthologs or proteins of similar function were found in *S. cerevisiae* screens with this compound, again underscoring the need for direct study of *C. albicans* to identify novel treatment strategies.

The approach of using tagged transposon mutagenesis to generate mutant collections can be applied to a wide range of fungi of medical interest. The *in vitro* mutagenesis method allows flexibility because organism-specific transposons are not required, although a means for homologous recombination is needed. However, this approach could also be adapted to an *in vivo* format if the transposon could be electroporated directly into the cell (e.g., using commercially available transposome technology [54]), or if it could be expressed endogenously. Both of these approaches could bypass the transformation step, although they may be subject to insertion bias. Additionally, the TagModules can be readily adapted to a targeted deletion system for fungi with a compatible recombination system. While model organisms such as *S. cerevisiae* have been invaluable in initiating research in a range of microorganisms of medical interest, ultimately, it will be most fruitful to identify novel treatment strategies using the pathogen itself, owing to pathogen-specific differences. In summary, we have generated a uniquely tagged, publically available and archived disruption collection in *C. albicans* that can be used in multiplexed phenotypic assays or in individual experiments to identify potential new biology, therapeutic targets and mechanisms of pathogenesis.

## Methods

All primers, plasmids, and strains are detailed in Tables S9, S10, and S11. Additional details are available on the supplementary website (http://chemogenomics.med.utoronto.ca/supplemental/transposon/) and as a text file (Methods S1). GO analysis (http://www.candidagenome.org/cgi-bin/GO/goTermFinder) was performed between 01/2010-02/2010.

### Library construction

*C. albicans* genomic DNA was isolated from strain BWP17 (*ura3*Δ*::* λ *imm434/ura3*Δ*::*λ*imm434 his1::hisG/his1::hisG arg4::hisG/arg4::hisG)* [55] and partially digested with one or more of the following enzymes: XbaI, EcoRV, SpeI, XbaI/SpeI, XbaI/EcoRV, or BsrBI. The partially digested DNA fragments were gel purified to approximately 2–8 kb in size and ligated into a library backbone cut with the corresponding enzyme and phosphatase-treated. Backbones used were pCR 8/GW/TOPO + linker for XbaI, EcoRV, and BsrBI-cut genomic DNA, and pUC19 + linker for SpeI, XbaI/EcoRV, and SpeI/XbaI-cut genomic DNA. Construction of these vectors was previously described [27]. Each library contained on average 20,000 clones. We also used a commercially available *C. albicans* genomic library (Open Biosystems; [56]).

### Transposon mutagenesis

The transposon destination vectors Tn7-*UAU1*-A and Tn5-*UAU1*-C.1, modified from Tn7-*UAU1* [21] and EZ-Tn5 pMOD-3 (Epicentre Biotechnologies) to contain the Gateway recombination sites, were obtained from our previous study [27]. The Gateway-compatible TagModules [27] were pooled and transferred to the Tn7-*UAU1*-A or the Tn5-*UAU1*-C.1 vectors as previously described [27], and the resulting tagged transposons were used in the mutagenesis of *C. albicans* genomic libraries as described in the same study. The majority of insertions were generated using the Tn5-*UAU1* due to technical difficulties with the Tn7. Each insertion was sequenced using D2_revcomp (Tn7-based) or U1 (Tn5-based) to identify the gene disrupted and the tag associated with each disruption. Gene-tag pairs were sorted to maximize i) the number of unique TagModules, ii) unique gene mutagenesis events, and iii) highest % gene disrupted (# base pairs from transposon junction to gene start)/(total gene length). Overall genome coverage is represented in Figure S1. Select insertion plasmids were then plasmid prepped with Seqprep 96 (Edge Biosystems), and the genomic fragment containing the tagged insertion was excised with the appropriate enzyme and chemically transformed into BWP17 in 96-well format.

At this step, two pools were created, one with unique uptags and one with unique downtags (for details, see Figure S2 and Methods S1; for pool characteristics, see Table S1). We separately arrayed transformants for each pool to agar plates and scraped the colonies into SC- Arg + uridine +15% glycerol to create the *C. albicans* pools, which we then stored as 50 µL aliquots at −80°C. For validation, we grew the pools independently in YPD for 20 generations as described in [34] minus the 10-generation recovery time, and extracted genomic DNA using the YeaStar Genomic DNA Kit (Zymo Research). PCR amplification of the uptags and downtags was performed separately with the common primers U1′ & BTEG-U2′, and D1′ & BTEG-D2′, and 30 µL each of uptag and downtag product was hybridized to an Affymetrix TAG4 microarray as described [57].

### Pooled growth assays

For haploinsufficiency experiments, YPD, SC – Arg + uridine, YNB + uridine, and SLAD + uridine were made as defined in [58]. Growth assays were performed in duplicate in each of the media conditions, and samples were recovered at 5, 10, 15, and 20 generations of growth. Genomic DNA extraction, tag amplification, and hybridization were performed as described above. In total, we performed a series of eight hybridizations per media condition plus two hybridizations for a common zero timepoint. For the zero timepoint, ∼2 OD_600_ of frozen cells were used as the cell template for the genomic extraction.

For array data pre-processing, we followed the protocol outlined in [57], [59]. Briefly, outliers were masked and removed, and the average of the unmasked replicates were calculated for each tag. Uptags and downtags were mean-normalized, and low-quality tags (those below 3X background intensity units) were removed. To identify strains with reduced fitness in each of these growth conditions, we used a linear regression model to track decreases in tag hybridization intensity as a function of time. Linear regression of the log_2_(tag intensity) as a function of generations of growth (0, 5, 10, 15, and 20) was implemented using the lm() function in the statistical program R. A negative regression slope indicates that the tag signal decreases over time, inferring that the strain has a fitness defect in this condition with respect to the pool as a whole. To correct for multiple testing, the p-value for each regression was adjusted using the false discovery rate (“fdr”) option of the R function p.adjust(). For comparisons of regression slope between media types, an F-statistic was calculated and a p-value derived from the distribution. Our criteria for strain sensitivity was slope <0, p<0.05. This is a slightly less stringent cutoff than reported in Deutschbauer et al. (2005) as we have observed that the competitive growth assay is capable of detecting even slight defects in growth.

### Complementation testing

For complementation testing, we used several pre-existing *S. cerevisiae* resources for the strain background. We picked individual strains from the Magic Marker collection (Open Biosystems, [32]) of yeast heterozygous deletions containing the selectable matA haploid marker. As the Magic Marker strains have some reversion rate, as evidenced by some colonies observed on the no-vector plates, we confirmed a few of these results with traditional tetrad dissection of the sporulated yeast knockout (YKO) strain, which has no Magic Marker cassette. For overexpression, we used as our destination vector the Gateway compatible destination vector pAG416GPD-ccdB (Addgene, [60]), which is a Ura+ CEN plasmid under control of a constitutive promoter.

For cloning *C. albicans* ORFs, we PCR amplified approximately 500 bp up and downstream of the start codon using primers specific to each gene and Platinum PCR SuperMix High Fidelity Primer Solution (Invitrogen). PCR products were TOPO cloned into the Gateway entry vector pCR 8/GW/TOPO (Invitrogen). Individual clones were sequenced using primers GW1 and GW2. Correct clones were then transferred to the pAG416GPD-ccdB destination vector using the LR clonase reaction (Invitrogen), and resequenced as described. For *S. cerevisiae* overexpression plasmids, we picked clones from the Molecular Barcoded Yeast (MoBY) ORFs (Open Biosystems, [61]). As these were already Gateway compatible, we transferred them to the overexpression plasmids using the LR clonase reaction and sequence verified them using primers GPD_ProF and pBluescriptSK.

Overexpression plasmids were chemically transformed into the corresponding Magic Marker or YKO strain, selecting for Ura+ transformants. Individual colonies were then inoculated to SC –Ura and grown overnight. Cultures were then harvested, washed twice with water, and then resuspended in sporulation media (2% potassium acetate) at a density of 1–1.5 OD_600_/mL. After sporulation for 5 days, 4.5×10^−4^ OD_600_ of sporulated Magic Marker culture was plated to Magic Marker selection media –Ura and grown for 2–3 days at 30°C. YKO-based strains were tetrad dissected and plated onto SC –Ura + G418. Tetrad dissected plates were then replica plated onto 5-FOA and YPD + G418 agar plates to confirm complementation.

### Individual growth experiments

For individual growth experiments, we picked mutants from the frozen stock and grew them to saturation in SC –Arg + uridine. Each strain was then diluted to a working OD_600_ of ∼0.6 in water. For 5 generation experiments, each strain was diluted to a final OD_600_ of 0.06 in YPD, SC –Arg + uridine, YNB, or SLAD in 96-well plates. For 20 generation experiments, each strain was diluted to a final OD of 0.03 in the media of interest in 48-well plates, and growth was measured every 15 minutes robotically diluting every 5 generations as in the pooled growth assay. To measure growth of the GRACE strains, 12 of 17 of the core haploinsufficient strains identified in our screens were obtained as GRACE alleles (5 were not constructed), in which heterozygotes were constructed such that the remaining allele is under the control of a tetracycline-repressible promoter [33]. 400 cells of each strain (as determined by hemocytometry) were inoculated into 650 µL of media in 48-well plates (Greiner) and grown with constant shaking at 30°C as described [57]. In these conditions, individual cultures underwent ∼14 generations of growth without necessitating re-inoculation during the course of the experiment. Each strain was assayed in triplicate in selective SC –Arg + uridine media in the presence or absence of the tetracycline analog doxycycline (100 µM). AvgG (a metric of growth) was calculated as previously described [34].

### Test for filamentation on solid media

Individual strains were picked from the frozen stock and grown overnight in SC –Arg + uridine. Strains were then stamped in triplicate to Spider agar media and grown for 5 days at 30°C, and colonies were then examined microscopically for filamentation. Methods for the pooled assay on solid SLAD media are described in Methods S1.

### Drug screening

A diversity library was obtained from ChemDiv, Inc. and dissolved in DMSO. Other compounds were obtained from Sigma with the exception of itavastatin ca (Sequoia Research Products) and enantio-paf C-16 (Enzo Life Sciences). Each compound had previously been titrated to an inhibitory level of ∼10% in *S. cerevisiae* in YPD buffered to a pH of 6.8 with HEPES [62]. We grew *C. albicans* strain BWP17 and *S. cerevisiae* strain HO-1 in the presence of these 1521 distinct compounds under the same conditions, measuring AvgG as previously described [34]. Titration experiments were performed in microtitre plates, growing BWP17 and HO-1 in 100 µL buffered YPD plus either 1% DMSO (control) or 2 µL of compound at stock concentration. Growth rate in compound dilutions of up to 1/64^th^ original concentration (or further, if necessary) were measured as previously described. AvgG from each microtitre plate was normalized to the 12 controls on each plate.

Selected compounds were then chosen for follow-up in dose response and pooled growth assays. First, compounds were ranked in order of greatest difference in inhibition between *C. albicans* and *S. cerevisiae* (Δ(normalized AvgG)). As a number of the compounds which produced the greatest difference in inhibition were not readily available or obtainable, and some hybridizations in the pooled growth assay failed, we focused on ∼67 available compounds for follow-up. The majority of these produced greater inhibition of *C. albicans* in the dose response assays (Figure S6). For pooled growth assays with 57/67 of these compounds, 20-generation pooled growth assays were performed as described [34], [62]. To determine sensitive strains, the experimental array was compared to a matched control set comprised of 11 no-drug arrays [18]. We then calculated z-scores and associated p-values as a metric of sensitivity as described [18].

## Supporting Information

Methods S1 Supplementary methods.
(0.07 MB DOC)

Figure S1 Multiple genomic libraries are necessary to improve genome coverage. (A) Transposon insertion sites are plotted by coordinate organized by chromosome. We used one commercial library (blue) and constructed a total of six genomic libraries. Green: genomic DNA was digested to completion with XbaI, yellow: SpeI, black/cyan: genomic DNA was partially digested with XbaI/SpeI or XbaI/EcoRV; red: remaining libraries were generated by digestion of genomic DNA with either EcoRV or BsrBI. The majority of mutants were generated using either XbaI- or SpeI-generated genomic libraries. (B) Close-up of terminal region of chromosome 8; colors are as in (A).
(0.96 MB TIF)

Figure S2 Scheme and validation for using one tag per strain. (A) Unique uptags were selected for one pool and unique downtags for a second pool, overall representing 4401 strains (4388 unique genes). The two pools are then screened and tags amplified in parallel to prevent cross-contamination of overlapping tags. The uptag and downtag PCRs can then be combined prior to hybridization. (B) Validation of the one tag per strain approach outlined in Figure S2A. Two uniquely tagged pools were used to increase the number of strains able to be represented on an array. Hybridization performance of the pool was compared to see if strain tracking was affected depending on whether the uptag or a downtag was used to represent a strain. Two independent pools (“pool 1” and “pool 2”) of the 4252 successfully transformed strains were grown for 20 generations in YPD +1% DMSO. Uptags were amplified from pool 1, and downtags from pool 2, and hybridized to a TAG4 array. In a “tag swap”, downtags from pool 1 and uptags from pool 2 were then amplified and hybridized to an array. Pearson correlation of tag intensities above 3X background is indicated in the upper left corner.
(0.71 MB TIF)

Figure S3 Cross-hybridization of tagged strains to unused tags. Distribution of log_2_ tag intensities of the remaining 12686 unused tags on the array. Log_2_ of background intensity  =  ∼6; cutoff for detection was 3X background (log_2_  =  ∼7). 394 tags were above 3X background; 166 corresponded to repaired tags [6], which have significant sequence similarity to other tags, 116 with no sequence similarity, and 112 corresponded to unused TagModules.
(0.27 MB TIF)

Figure S4 Confirmation of haploinsufficient phenotype with GRACE strains. (A) The 17 “core” strains were monitored for growth over 20 population doublings in a microplate reader in triplicate, with representative curves shown. Every 5 generations, cells were robotically transferred to a well containing fresh media. Growth data from the 2nd (∼10 generations), 3rd (∼15 generations), or 4th (∼20 generations) transfers was plotted against time (for some mutants, 15 or 20 generation growth data was not available). In all plots, black represents wild-type BWP17; each plot represents the mutants grown in a single plate with its own wild-type control. All curves were grown in selective SC media. (B) The 12 GRACE strains [8] were grown in selective SC media over ∼10–15 generations of growth. In (C), these mutants were grown in the presence of 100 µM doxycycline. In (D), the mean of triplicate AvgGs as percentage of wild-type growth were calculated for each curve. As growth curves in (A) were performed via robotic transfer, we were unable to calculate AvgGs for these curves.
(1.21 MB TIF)

Figure S5 Additional complementation tests of *C. albicans* ORFs. Description is as in Figure 4. All negative results with the Magic Marker strains (top round panels) were confirmed by tetrad dissection (representative tetrads are in bottom rectangular panels, if applicable). Top panel (round plates): left, negative control (vector-only); center, complementation with *C. albicans* ORF; right, positive control (complementation with corresponding *S. cerevisiae* ORF). Bottom panels (rectangular): all tetrads were replica-plated onto media containing 5-fluoroorotic acid (5-FOA) or geneticin (G418) to confirm that the overexpression clone was the source of complementation. First row: the tetrad dissection (left is negative control (vector-only); center, complementation with *C. albicans* ORF; right, positive control (complementation with corresponding *S. cerevisiae* ORF)). Second row: replica plate to 5-FOA; third row: replica plate to geneticin (G418).
(9.80 MB TIF)

Figure S6 Additional dose response curves. Description is as in Figure 6.
(1.44 MB TIF)

Figure S7 Chemical structures of compounds screened in the pooled growth assay.
(2.74 MB TIF)

Figure S8 Distribution of pair-wise compound similarity. Compound similarity was calculated based on an ECFP_4 representation of each compound and scored with the Tanimoto coefficient. The distribution for the 57 compounds screened is shown in red. A random set of 57 FDA compounds (blue) is also shown as a comparison. Both sets of compounds are structurally diverse, as the pair-wise similarity is lower than the widely used threshold of 0.3 to define diversity when ECFP_4/Tanimoto is used [9].
(0.30 MB TIF)

Table S1 Summary of *C. albicans* mutants created
(0.02 MB XLS)

Table S2 List of heterozygous disruption *C. albicans* mutants generated in this study
(1.25 MB XLS)

Table S3 Regression slopes and adjusted p-values for all strains
(1.09 MB XLS)

Table S4 Genes haploinsufficient in all conditions
(0.03 MB XLS)

Table S5 Genes haploinsufficient in 3 or more conditions
(0.04 MB XLS)

Table S6 Genes haploinsufficient in hyphae-inducing conditions
(0.04 MB XLS)

Table S7 Z-scores and p-values for compound screens
(8.88 MB XLS)

Table S8 Strains defective in agar invasion from pooled assay in solid SLAD media
(0.09 MB XLS)

Table S9 Primers used in this study
(0.03 MB XLS)

Table S10 Strains used in this study
(0.03 MB XLS)

Table S11 Plasmids used in this study
(0.02 MB XLS)
