# Supplementary material for: Gene Annotation and Drug Target Discovery in Candida albicans with a Tagged Transposon Mutant Collection
Source: PLoS Pathog. 2010 Oct 7;6(10):e1001140. doi: 10.1371/journal.ppat.1001140 (PMC2951378; doi:10.1371/journal.ppat.1001140)
Supplement: Methods S1 — Supplementary methods. (0.07 MB DOC) [file ppat.1001140.s001.doc]

**­Methods S1**

**Additional details on the creation and validation of the tagged *C. albicans* collection.**

For the mutagenesis, we used an EZ-Tn5 (Epicentre) transposon and modified it to contain i) the Gateway compatibility cassette for the TagModule transfer, ii) a kanamycin resistance marker for selection of insertion events in *E. coli*, and iii) a *UAU1* selection cassette [1], which allows selection for integration into the *C. albicans* genome and can be used to generate heterozygous (Arg+) as well as homozygous disruption mutants (Arg+, Ura+). The final step to produce uniquely tagged transposons was to transfer the TagModules into the modified Tn5 vector. Because the Gateway reaction is highly efficient and specific ([2]; data not shown), TagModule transfer to the modified Tn5 transposon was performed in pools, resulting in pools of tagged transposons.

We used the pools of tagged transposons to mutagenize a commercial genomic library (Open Biosystems; [3]) *in vitro*. However, we found that we quickly saturated this library as few insertions in new genes were recovered after additional rounds of mutagenesis and sequencing. To improve genome coverage, we chose to use multiple, alternatively cloned genomic libraries, which were created by digesting *C. albicans* genomic DNA with different combinations of restriction enzymes. By using multiple libraries (thereby reducing cloning biases), we were able to improve coverage (Figure S1).

To transform the desired transposon insertion events into *C. albicans* to generate heterozygous alleles, the genomic fragment containing the transposon insertion was excised from the library. This flanking genomic sequence was then used for homologous recombination of the transposon insertion into the *C. albicans* genome. Because the average library insert size ranged from 2-8kb, we estimate that these homologous regions were generally well in excess of 60 bp, which typically yield transformation efficiencies of 97%+ in *S. cerevisiae* (A. Chu, personal communication). We performed a PCR-based test for integration using primers flanking regions from within the transposon to 611-726 bp outside the transposon insertion. Tests of two independent insertion events of *ERG11* showed a PCR product indicating integration for 15/15 and 13/15 colonies picked, respectively; we also tested integration of *ERB1* (14/15), and *CDC37* insertions (22/24). We are confident that these numbers represent the lower bounds for correct integration as we used a relatively crude colony PCR-based test. Because this PCR-based method tests only for homologous integration, it is possible that there may be additional ectopic integration of the transposon-containing genomic fragment. However, given the long homologous flanking regions contained within these fragments to mediate recombination, we expect that ectopic integration would be a rare event [4].

So that there would be no overlap of tagged strains when pooled, we sorted our 21468 insertion events to maximize the number of unique genes associated with unique TagModules. We found, however, that we had fewer unique TagModules than genes available; only 3838 unique TagModules were represented across the 4827 genes. Accordingly, we asked if using one tag of a TagModule (effectively doubling the number of usable tags) would provide sufficient discrimination of individual strains in a pool. This was supported by the previous observation that the performance of both the uptag and downtag in a TagModule was robust, quantitative, and correlated [5]. Under this approach, we created two pools, termed “pool 1” and “pool 2”. Each pool has a unique set of transposon insertions but contains an overlapping subset of TagModules. Therefore, we amplify only uptags from one pool, and only downtags from the second pool. For pooled growth assays, each pool is screened and tags amplified separately prior to combining the amplicons for microarray hybridization (Figure S2A). Using this one-tag approach, we were able to select 4401 uniquely tagged genes (representing 4388 unique genes) for transformation into *C. albicans* (Table S1, Table S2).

To validate that this one-tag approach was reproducible regardless of whether the uptag or the downtag was used to represent a strain, we performed a “tag swap” experiment (Figure S2B). We assigned “pool 1” unique uptags, and “pool 2” unique downtags, amplifying the tags separately but combining the products prior to hybridization to an array. We compared this to the hybridization of the reverse combination and found that strains with above 3X background intensity were significantly correlated (R= 0.64, p<10^-16^). As reported in the main text, biological replicates of this pool grown under twenty generations of growth in a pooled assay were highly correlated (Figure 2B; R= 0.98, p<10^-16^). These results indicate that the hybridization performance of a strain is similar regardless of whether it was represented by its uptag or its downtag and that little information on tag abundance is lost by using only one tag. For the remainder of this study, we report data on one tag per strain.

To validate that the tags were successfully incorporated into each mutant strain following transformation into *C. albicans*, we tested the hybridization performance of our tagged mutants by pooling them in 384-strain batches, amplifying the tags, and hybridizing them to an array. Of the 4401 attempted transformations, we recovered colonies for 4239 of these, based on our qualification that a strain’s tag must have signal intensity above 3X median background intensity (Table S2). As reported in the main text, when we pooled all of the 4239 strains, amplified their tags, and hybridized them to an array, we detected 3633 (85%) with tag intensity above 3X median background, with 619 strains falling below background (Figure 2C).

Finally, to validate that our strains did not have significant cross-reactivity with other features on the array (due to cross contamination, sequencing errors, or errors in sample tracking), we examined the signal intensities of all unused tags on the array. The vast majority, 12292 of 12686 (97%) fell at or below 3X background (Figure S3). Of the 394 above 3X background, 166 (1.3%) corresponded to previously repaired tags, which have significant sequence similarity to tags in use [6]. 116 (0.9%) corresponded to tags with no significant sequence similarity to the tags used to create the TagModules, and 112 (0.9%) corresponded to TagModule tags that were included in the initial pool of uniquely tagged transposons, but were not included in the subsequent mutant collection. This last number may represent a low level of error in sample tracking (e.g., an incorrect tag being assigned to a strain, or cross contamination in the collection); nonetheless, the majority of these have significantly lower signal intensity relative to the expected TagModules in our pool (one-tailed t-test, p<1.7x10^-12^).

**Pooled growth on solid SLAD media**

Solid SLAD media was prepared as follows: One liter of media contained 20 g electrophoresis-grade agarose, 1.7 g yeast nitrogen base (without ammonium sulfate and without amino acids), and 20 g glucose. Following autoclaving, filter-sterilized ammonium sulfate, histidine, and uridine were added to a final concentration of 50 μM, 10 μM, and 12.5 μM, respectively. 10 mL of media was then aliquotted to 85 mm Petri dishes. The two *C. albicans* pools were plated independently, in duplicate, for a total of ~500,000 colonies over 15 plates. Plates were sealed with parafilm and incubated at 37°C for six days, at which point samples of agarose were examined microscopically to verify cell invasion. 3 mL of water were then added to each plate, gently rubbed with a glove, and this non-invaded portion (“supernatant”) was collected. The plates were then washed under running tap water. The remaining agarose, which contained the invaded cells, was transferred to a beaker (100 g/beaker) and 200 mL of Qiagen QG solubilization buffer added. The agarose was melted for 90 min at 60°C, after which the cells were pelleted at 10,000 RCF in a Sorvall Centrifuge (“pellet”). Samples of the supernatant and pellet cells were processed and hybridized as described [7]. Log_2_ ratios (supernatant/pellet) were calculated from the mean-normalized data for each replicate and averaged.

**Supplementary Figures and Tables**

**Figure S1.** Multiple genomic libraries are necessary to improve genome coverage.

(A) Transposon insertion sites are plotted by coordinate organized by chromosome. We used one commercial library (blue) and constructed a total of six genomic libraries. Green: genomic DNA was digested to completion with XbaI, yellow: SpeI, black/cyan: genomic DNA was partially digested with XbaI/SpeI or XbaI/EcoRV; red: remaining libraries were generated by digestion of genomic DNA with either EcoRV or BsrBI. The majority of mutants were generated using either XbaI- or SpeI-generated genomic libraries.

(B) Close-up of terminal region of chromosome 8; colors are as in (A).

**Figure S2.** Scheme and validation for using one tag per strain.

(A) Unique uptags were selected for one pool and unique downtags for a second pool, overall representing 4401 strains (4388 unique genes). The two pools are then screened and tags amplified in parallel to prevent cross-contamination of overlapping tags. The uptag and downtag PCRs can then be combined prior to hybridization.

(B) Validation of the one tag per strain approach outlined in Figure S2A. Two uniquely tagged pools were used to increase the number of strains able to be represented on an array. Hybridization performance of the pool was compared to see if strain tracking was affected depending on whether the uptag or a downtag was used to represent a strain. Two independent pools (“pool 1” and “pool 2”) of the 4252 successfully transformed strains were grown for 20 generations in YPD + 1% DMSO. Uptags were amplified from pool 1, and downtags from pool 2, and hybridized to a TAG4 array. In a “tag swap”, downtags from pool 1 and uptags from pool 2 were then amplified and hybridized to an array. Pearson correlation of tag intensities above 3X background is indicated in the upper left corner.

**Figure S3.** Cross-hybridization of tagged strains to unused tags. Distribution of log_2_ tag intensities of the remaining 12686 unused tags on the array. Log_2_ of background intensity = ~6; cutoff for detection was 3X background (log_2_ = ~7). 394 tags were above 3X background; 166 corresponded to repaired tags [6], which have significant sequence similarity to other tags, 116 with no sequence similarity, and 112 corresponded to unused TagModules.

**Figure S4.** Confirmation of haploinsufficient phenotype with GRACE strains.

(A) The 17 “core” strains were monitored for growth over 20 population doublings in a microplate reader in triplicate, with representative curves shown. Every 5 generations, cells were robotically transferred to a well containing fresh media. Growth data from the 2^nd^ (~10 generations), 3^rd^ (~15 generations), or 4^th^ (~20 generations) transfers was plotted against time (for some mutants, 15 or 20 generation growth data was not available). In all plots, black represents wild-type BWP17; each plot represents the mutants grown in a single plate with its own wild-type control. All curves were grown in selective SC media.

(B) The 12 GRACE strains [8] were grown in selective SC media over ~10-15 generations of growth. In (C), these mutants were grown in the presence of 100 μM doxycycline. In (D), the mean of triplicate AvgGs as percentage of wild-type growth were calculated for each curve. As growth curves in (A) were performed via robotic transfer, we were unable to calculate AvgGs for these curves.

**Figure S5.** Additional complementation tests of *C. albicans* ORFs. Description is as in **Figure 4**. All negative results with the Magic Marker strains (top round panels) were confirmed by tetrad dissection (representative tetrads are in bottom rectangular panels, if applicable).

Top panel (round plates): left, negative control (vector-only); center, complementation with *C. albicans* ORF; right, positive control (complementation with corresponding *S. cerevisiae* ORF).

Bottom panels (rectangular): all tetrads were replica-plated onto media containing 5-fluoroorotic acid (5-FOA) or geneticin (G418) to confirm that the overexpression clone was the source of complementation. First row: the tetrad dissection (left is negative control (vector-only); center, complementation with *C. albicans* ORF; right, positive control (complementation with corresponding *S. cerevisiae* ORF)).

Second row: replica plate to 5-FOA; third row: replica plate to geneticin (G418).

**Figure S6.** Additional dose response curves. Description is as in **Figure 6.**

**Figure S7.** Chemical structures of compounds screened in the pooled growth assay.

**Figure S8.** Distribution of pair-wise compound similarity. ­

**Table S1.** Summary of *C. albicans* mutants created

**Table S2.** List of heterozygous disruption *C. albicans* mutants generated in this study

**Table S3.** Regression slopes and adjusted p-values for all strains

**Table S4.** Genes haploinsufficient in all c­onditions

**Table S5.** Genes haploinsufficient in 3 or more conditions

**Table S6.** Genes haploinsufficient in hyphae-inducing conditions

**Table S7.** Z-scores and p-values for compound screens

**Table S8.** Strains defective in agar invasion from pooled assay in solid SLAD media

**Table S9.** Primers used in this study

**Table S10.** Strains used in this study

**Table S11.** Plasmids used in this study

**References cited in all Supporting Information (Methods S1, Supplementary Figures, and Supplementary Tables)**

1. Enloe B, Diamond A, Mitchell AP (2000) A single-transformation gene function test in diploid *Candida albicans*. J Bacteriol 182: 5730-5736.

2. Gillette WK, Esposito D, Frank PH, Zhou M, Yu LR, et al. (2005) Pooled ORF expression technology (POET): using proteomics to screen pools of open reading frames for protein expression. Mol Cell Proteomics 4: 1647-1652.

3. Kadosh D, Johnson AD (2001) *Rfg1*, a protein related to the *Saccharomyces cerevisiae* hypoxic regulator *Rox1*, controls filamentous growth and virulence in *Candida albicans*. Mol Cell Biol 21: 2496-2505.

4. Gola S, Martin R, Walther A, Dunkler A, Wendland J (2003) New modules for PCR-based gene targeting in *Candida albicans*: rapid and efficient gene targeting using 100 bp of flanking homology region. Yeast 20: 1339-1347.

5. Oh J, Fung E, Price MN, Dehal PS, Davis RW, et al. (2010) A universal TagModule collection for parallel genetic analysis of microorganisms. Nucleic Acids Res 38: e146.

6. Pierce SE, Fung EL, Jaramillo DF, Chu AM, Davis RW, et al. (2006) A unique and universal molecular barcode array. Nat Methods 3: 601-603.

7. Pierce SE, Davis RW, Nislow C, Giaever G (2007) Genome-wide analysis of barcoded *Saccharomyces cerevisiae* gene-deletion mutants in pooled cultures. Nat Protoc 2: 2958-2974.

8. Roemer T, Jiang B, Davison J, Ketela T, Veillette K, et al. (2003) Large-scale essential gene identification in *Candida albicans* and applications to antifungal drug discovery. Mol Microbiol 50: 167-181.

9. Young DW, Bender A, Hoyt J, McWhinnie E, Chirn GW, et al. (2008) Integrating high-content screening and ligand-target prediction to identify mechanism of action. Nat Chem Biol 4: 59-68.

10. Davis DA, Bruno VM, Loza L, Filler SG, Mitchell AP (2002) *Candida albicans* Mds3p, a conserved regulator of pH responses and virulence identified through insertional mutagenesis. Genetics 162: 1573-1581.

11. Pan X, Yuan DS, Xiang D, Wang X, Sookhai-Mahadeo S, et al. (2004) A robust toolkit for functional profiling of the yeast genome. Mol Cell 16: 487-496.

12. Winzeler EA, Shoemaker DD, Astromoff A, Liang H, Anderson K, et al. (1999) Functional characterization of the *S. cerevisiae* genome by gene deletion and parallel analysis. Science 285: 901-906.

13. Alberti S, Gitler AD, Lindquist S (2007) A suite of Gateway cloning vectors for high-throughput genetic analysis in *Saccharomyces cerevisiae*. Yeast 24: 913-919.

14. Hu Y, Rolfs A, Bhullar B, Murthy TV, Zhu C, et al. (2007) Approaching a complete repository of sequence-verified protein-encoding clones for *Saccharomyces cerevisiae*. Genome Res 17: 536-543.
