# Supplementary figures and images for: Gene Annotation and Drug Target Discovery in Candida albicans with a Tagged Transposon Mutant Collection
Source: PLoS Pathog. 2010 Oct 7;6(10):e1001140. doi: 10.1371/journal.ppat.1001140 (PMC2951378; doi:10.1371/journal.ppat.1001140)

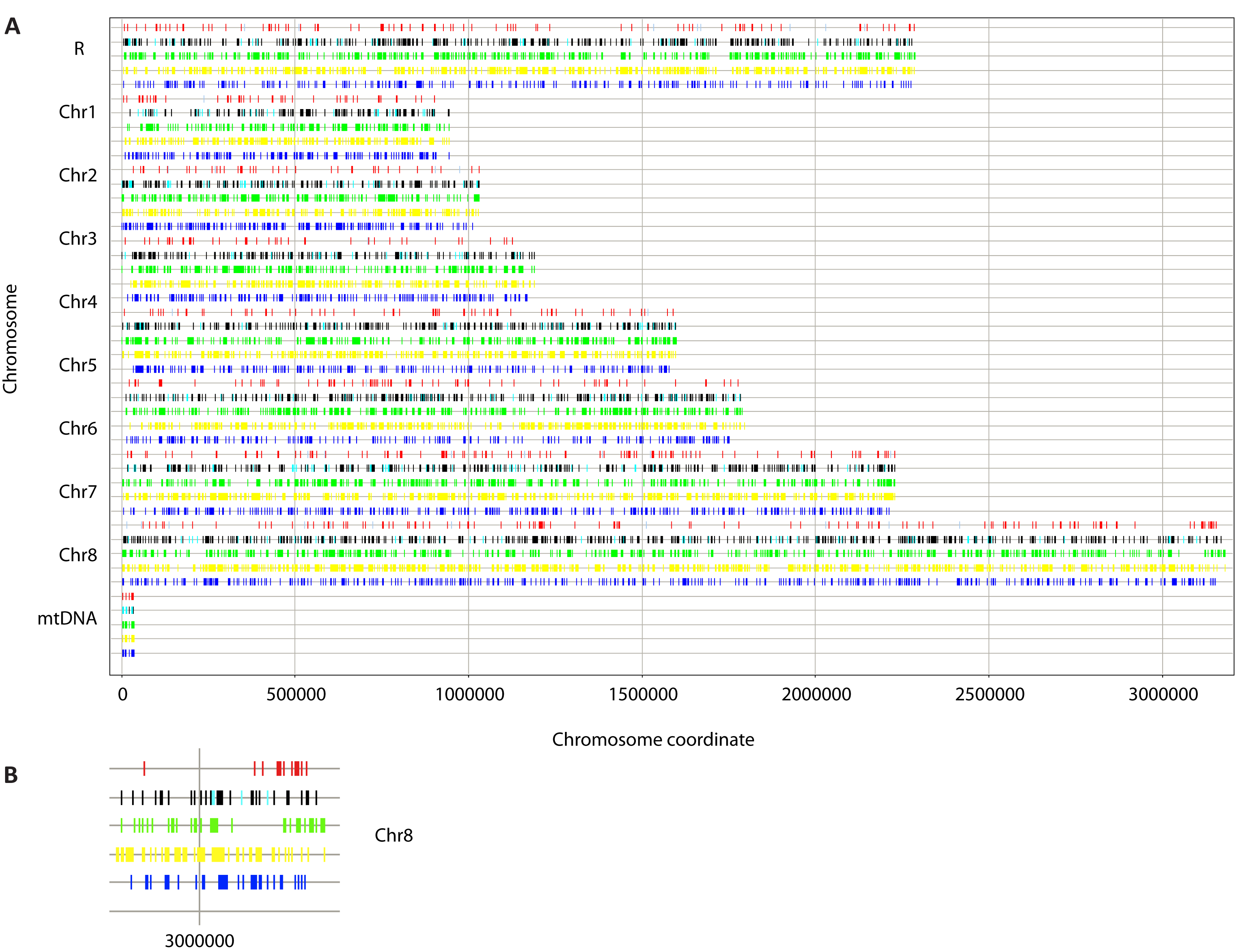

Supplement: Figure S1 — Multiple genomic libraries are necessary to improve genome coverage. (A) Transposon insertion sites are plotted by coordinate organized by chromosome. We used one commercial library (blue) and constructed a total of six genomic libraries. Green: genomic DNA was digested to completion with XbaI, yellow: SpeI, black/cyan: genomic DNA was partially digested with XbaI/SpeI or XbaI/EcoRV; red: remaining libraries were generated by digestion of genomic DNA with either EcoRV or BsrBI. The majority of mutants were generated using either XbaI- or SpeI-generated genomic libraries. (B) Close-up of terminal region of chromosome 8; colors are as in (A). (0.96 MB TIF) [file ppat.1001140.s002.tif]

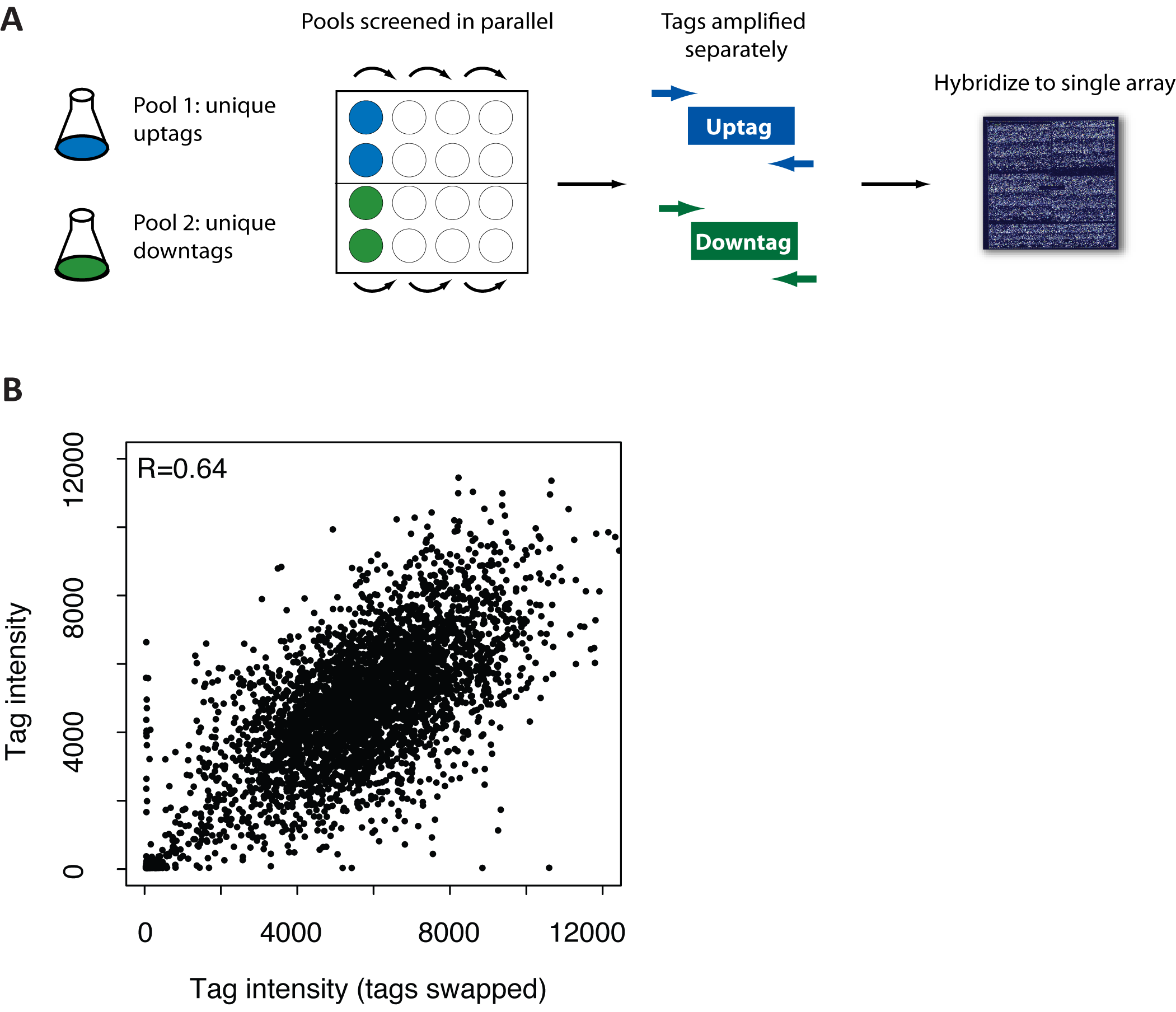

Supplement: Figure S2 — Scheme and validation for using one tag per strain. (A) Unique uptags were selected for one pool and unique downtags for a second pool, overall representing 4401 strains (4388 unique genes). The two pools are then screened and tags amplified in parallel to prevent cross-contamination of overlapping tags. The uptag and downtag PCRs can then be combined prior to hybridization. (B) Validation of the one tag per strain approach outlined in Figure S2A. Two uniquely tagged pools were used to increase the number of strains able to be represented on an array. Hybridization performance of the pool was compared to see if strain tracking was affected depending on whether the uptag or a downtag was used to represent a strain. Two independent pools (“pool 1” and “pool 2”) of the 4252 successfully transformed strains were grown for 20 generations in YPD +1% DMSO. Uptags were amplified from pool 1, and downtags from pool 2, and hybridized to a TAG4 array. In a “tag swap”, downtags from pool 1 and uptags from pool 2 were then amplified and hybridized to an array. Pearson correlation of tag intensities above 3X background is indicated in the upper left corner. (0.71 MB TIF) [file ppat.1001140.s003.tif]

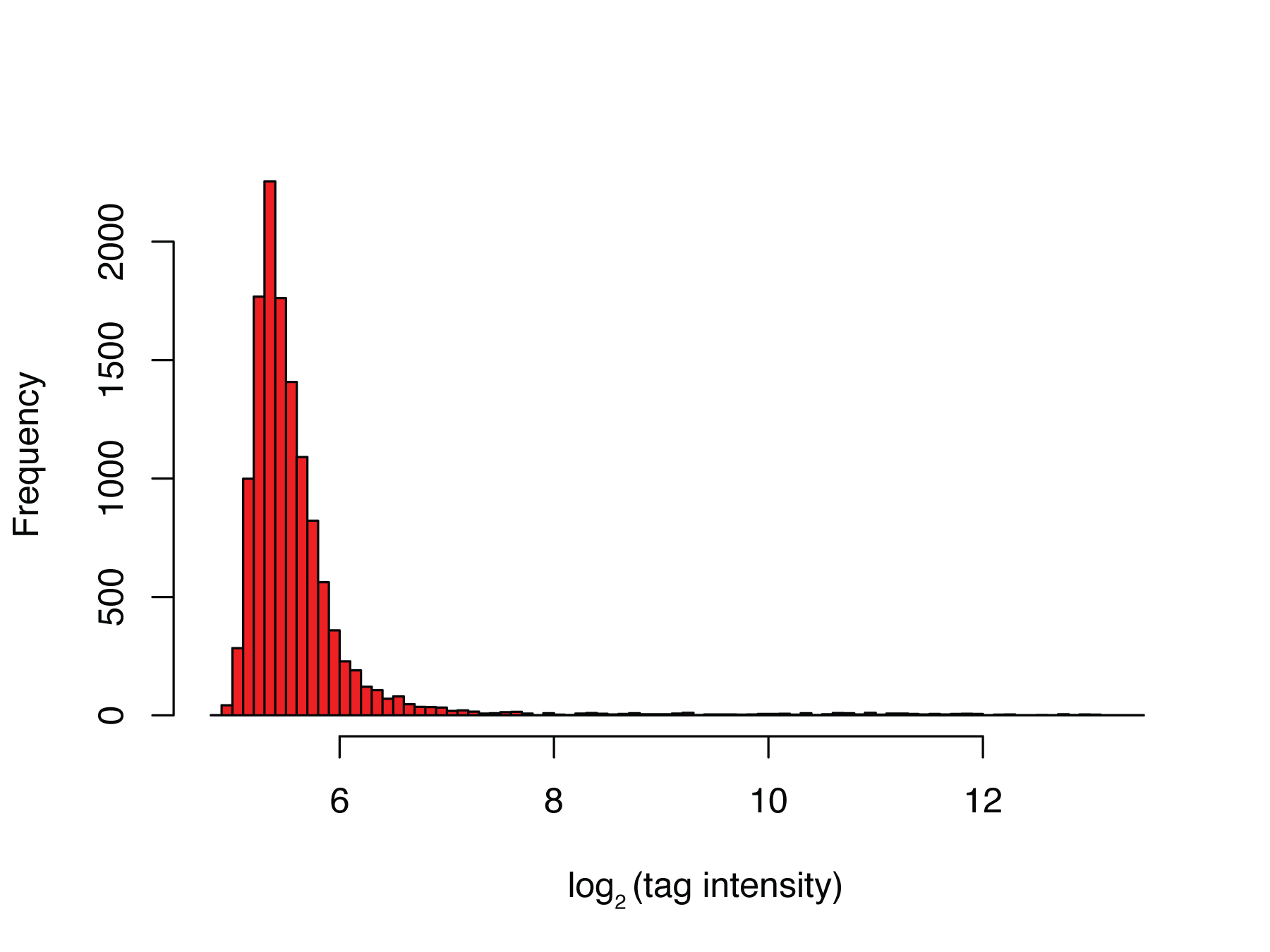

Supplement: Figure S3 — Cross-hybridization of tagged strains to unused tags. Distribution of log2 tag intensities of the remaining 12686 unused tags on the array. Log2 of background intensity = ∼6; cutoff for detection was 3X background (log2 = ∼7). 394 tags were above 3X background; 166 corresponded to repaired tags [6], which have significant sequence similarity to other tags, 116 with no sequence similarity, and 112 corresponded to unused TagModules. (0.27 MB TIF) [file ppat.1001140.s004.tif]

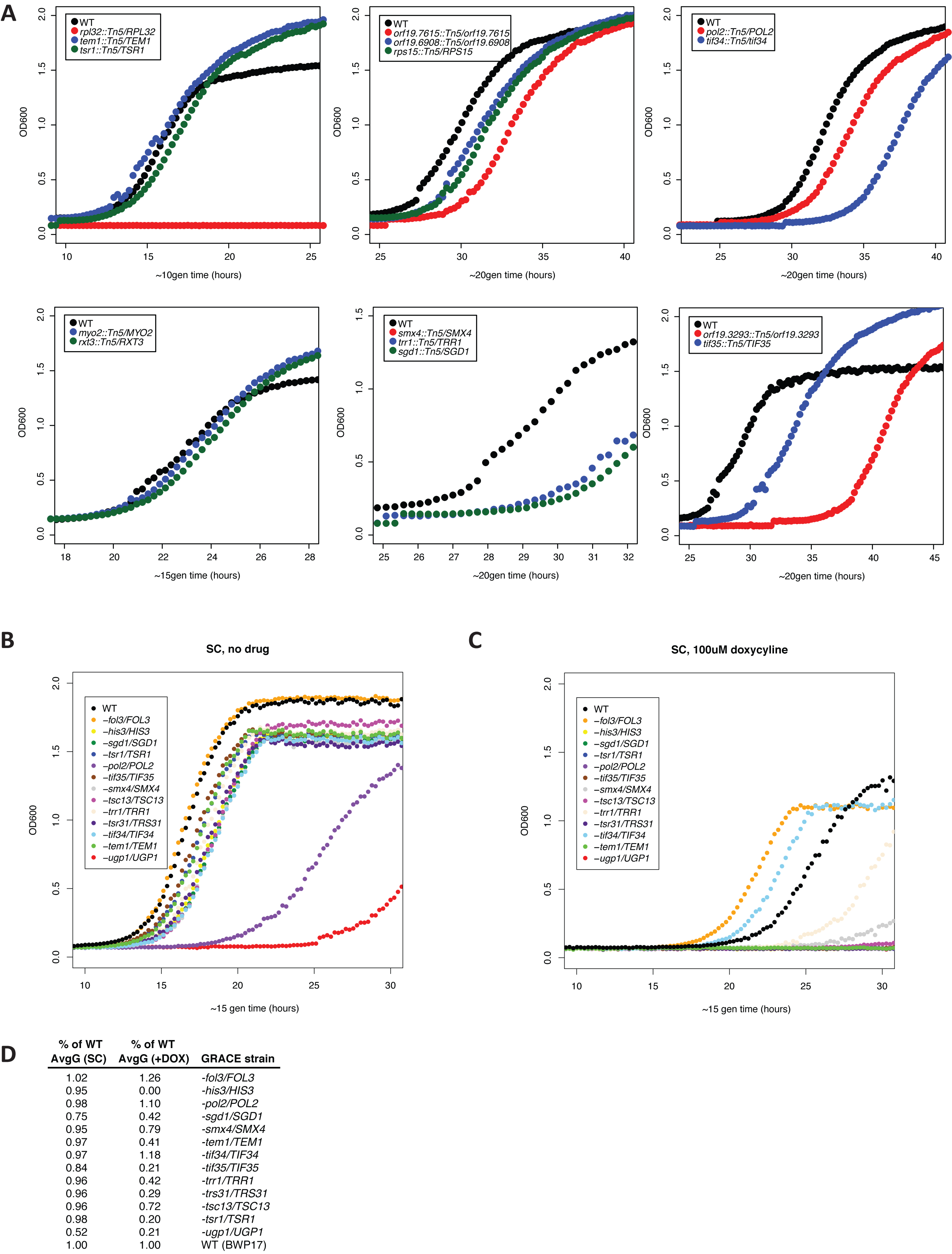

Supplement: Figure S4 — Confirmation of haploinsufficient phenotype with GRACE strains. (A) The 17 “core” strains were monitored for growth over 20 population doublings in a microplate reader in triplicate, with representative curves shown. Every 5 generations, cells were robotically transferred to a well containing fresh media. Growth data from the 2nd (∼10 generations), 3rd (∼15 generations), or 4th (∼20 generations) transfers was plotted against time (for some mutants, 15 or 20 generation growth data was not available). In all plots, black represents wild-type BWP17; each plot represents the mutants grown in a single plate with its own wild-type control. All curves were grown in selective SC media. (B) The 12 GRACE strains [8] were grown in selective SC media over ∼10–15 generations of growth. In (C), these mutants were grown in the presence of 100 µM doxycycline. In (D), the mean of triplicate AvgGs as percentage of wild-type growth were calculated for each curve. As growth curves in (A) were performed via robotic transfer, we were unable to calculate AvgGs for these curves. (1.21 MB TIF) [file ppat.1001140.s005.tif]

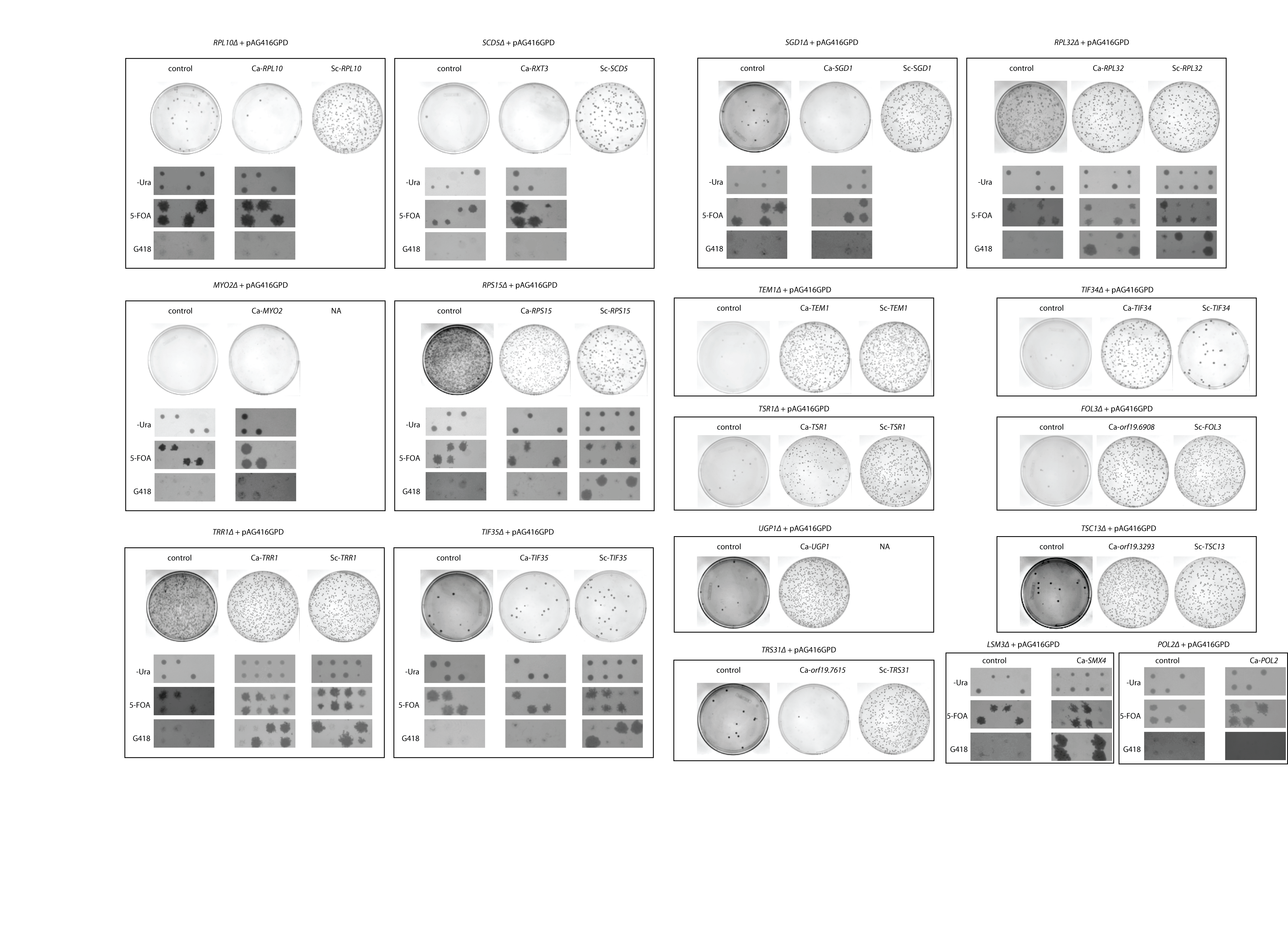

Supplement: Figure S5 — Additional complementation tests of C. albicans ORFs. Description is as in Figure 4. All negative results with the Magic Marker strains (top round panels) were confirmed by tetrad dissection (representative tetrads are in bottom rectangular panels, if applicable). Top panel (round plates): left, negative control (vector-only); center, complementation with C. albicans ORF; right, positive control (complementation with corresponding S. cerevisiae ORF). Bottom panels (rectangular): all tetrads were replica-plated onto media containing 5-fluoroorotic acid (5-FOA) or geneticin (G418) to confirm that the overexpression clone was the source of complementation. First row: the tetrad dissection (left is negative control (vector-only); center, complementation with C. albicans ORF; right, positive control (complementation with corresponding S. cerevisiae ORF)). Second row: replica plate to 5-FOA; third row: replica plate to geneticin (G418). (9.80 MB TIF) [file ppat.1001140.s006.tif]

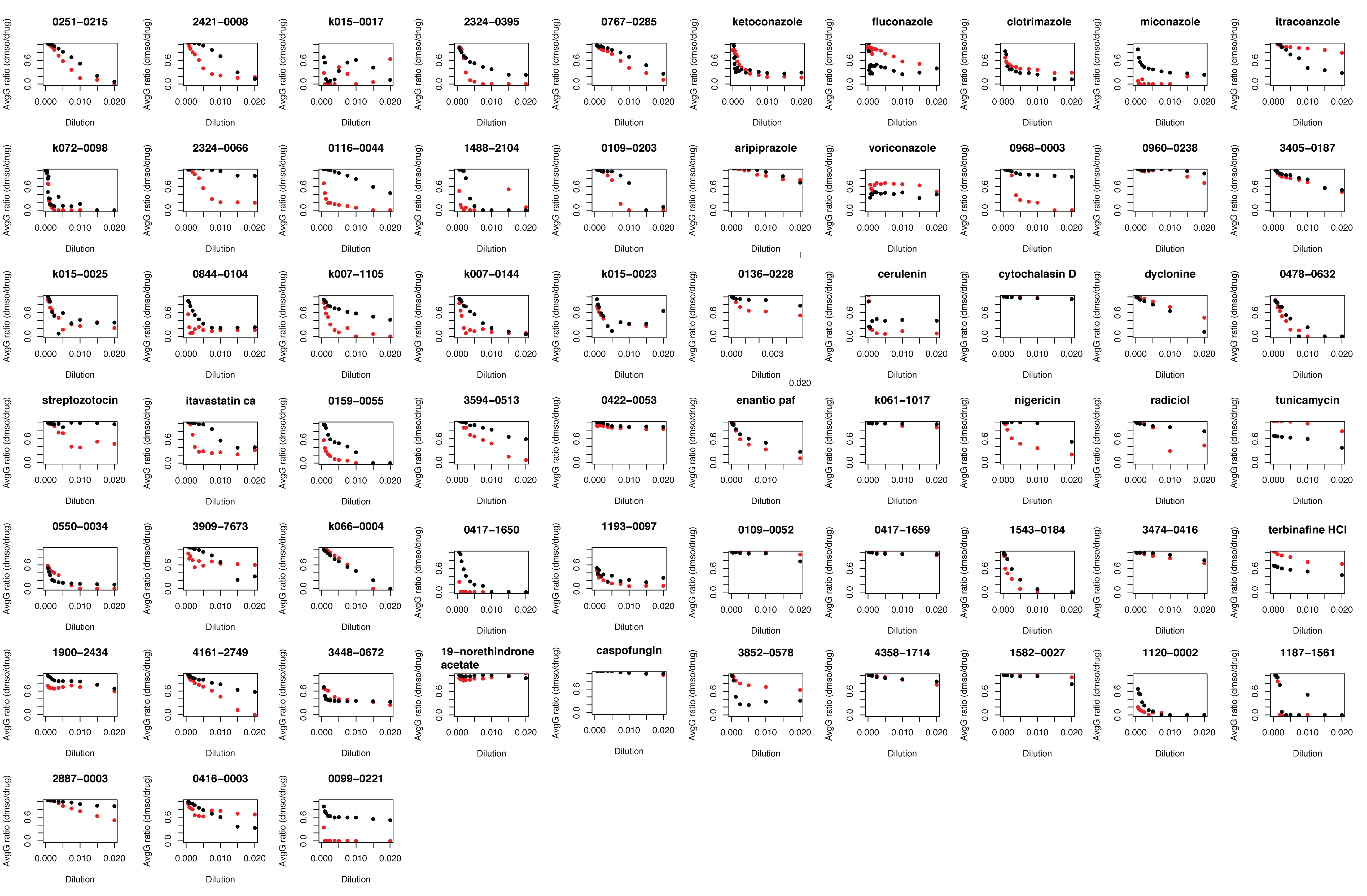

Supplement: Figure S6 — Additional dose response curves. Description is as in Figure 6. (1.44 MB TIF) [file ppat.1001140.s007.tif]

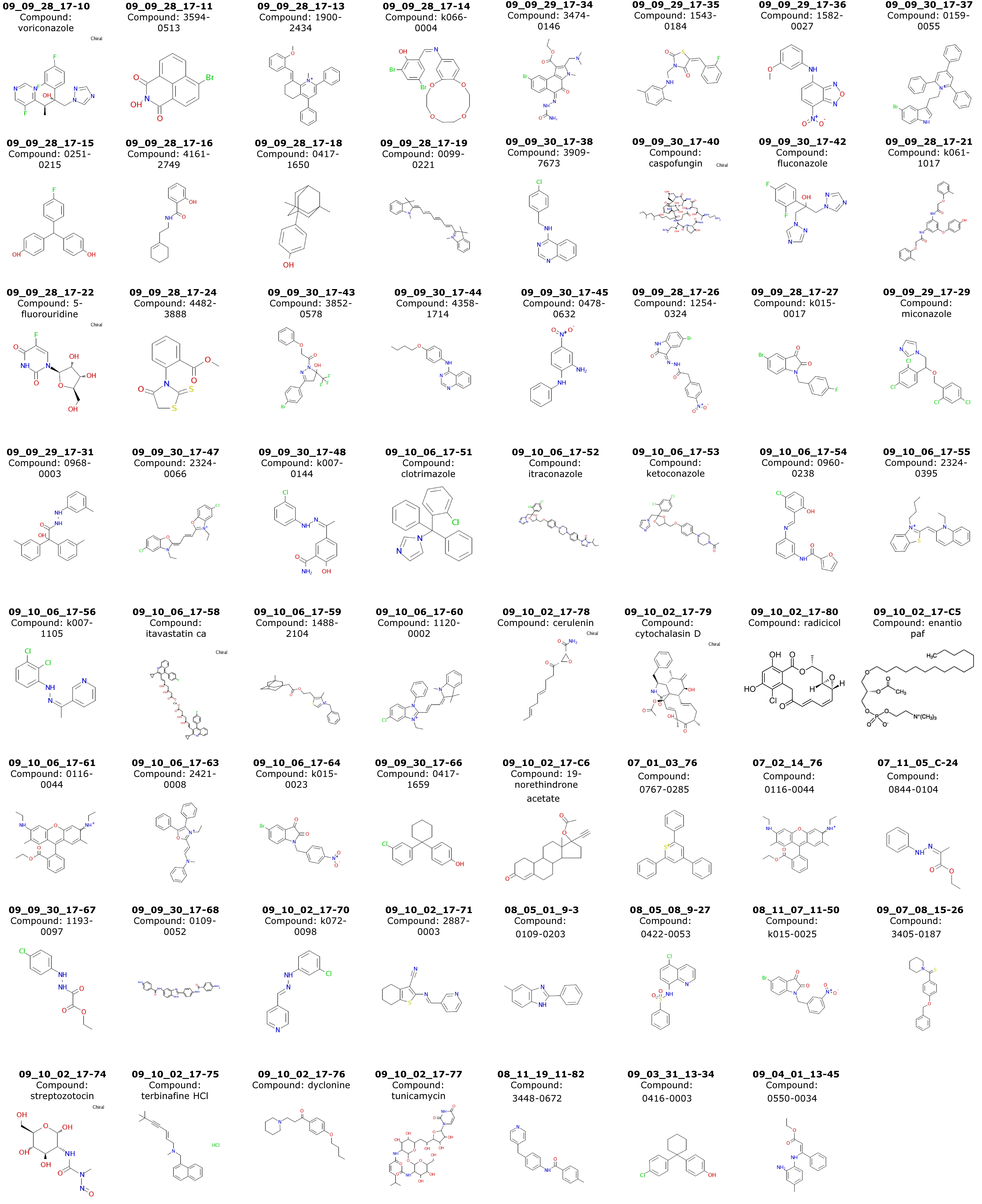

Supplement: Figure S7 — Chemical structures of compounds screened in the pooled growth assay. (2.74 MB TIF) [file ppat.1001140.s008.tif]

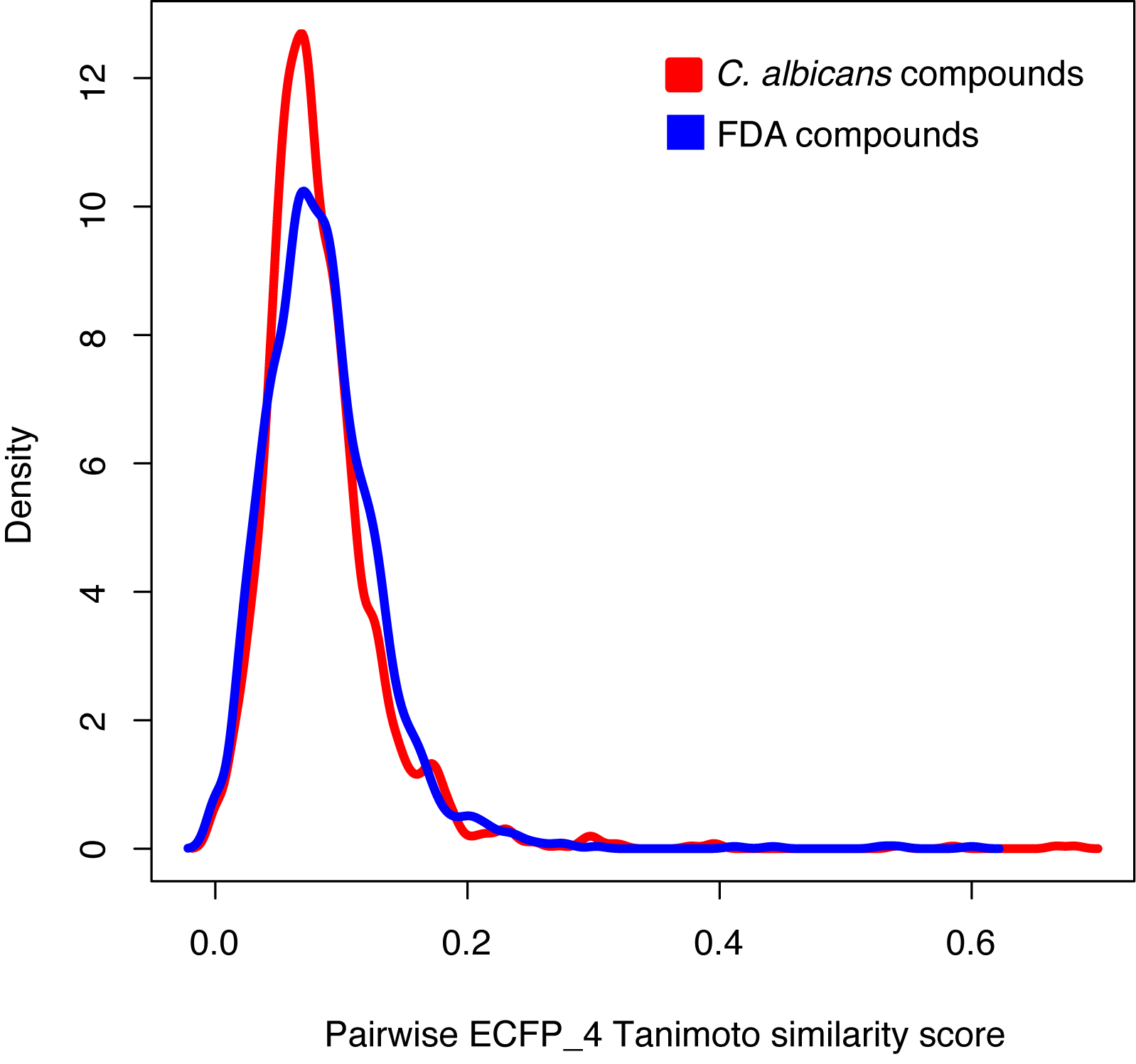

Supplement: Figure S8 — Distribution of pair-wise compound similarity. Compound similarity was calculated based on an ECFP_4 representation of each compound and scored with the Tanimoto coefficient. The distribution for the 57 compounds screened is shown in red. A random set of 57 FDA compounds (blue) is also shown as a comparison. Both sets of compounds are structurally diverse, as the pair-wise similarity is lower than the widely used threshold of 0.3 to define diversity when ECFP_4/Tanimoto is used [9]. (0.30 MB TIF) [file ppat.1001140.s009.tif]
